# *De novo* Transcriptome Analysis Revealed Genes Involved in Flavonoid and Vitamin C Biosynthesis in *Phyllanthus emblica* (L.)

**DOI:** 10.3389/fpls.2016.01610

**Published:** 2016-10-27

**Authors:** Avneesh Kumar, Sunil Kumar, Savita Bains, Vanya Vaidya, Baljinder Singh, Ravneet Kaur, Jagdeep Kaur, Kashmir Singh

**Affiliations:** Department of Biotechnology, Panjab UniversityChandigarh, India

**Keywords:** *Phyllanthus emblica*, flavonoids, vitamin C, transcriptome, gene ontology, simple sequence repeats, transcription factors

## Abstract

*Phyllanthus emblica* is an affluent source of various therapeutic components. A few of them like vitamin C and flavonoids are predominant bioactive compounds that are being used in immense pharmacological applications. In-spite of numerous applications, the genomic information of this plant was limited to a few expressed sequence tags (ESTs) in DNA databases. Herein, we developed in-depth transcriptome information of *P. emblica* using Illumina Hiseq 2000 platform and characterized. A total of 31,285,965 high-quality reads were assembled into 91,288 contigs with the N50 value 358. Out of them, 47,267 contigs were functionally annotated using BLASTX search against NCBI-non-redundant (NR) protein database. Further, 31,366 contigs showed similarity with various gene ontology (GO) terms, and 1299 were related to different enzymes and biosynthetic pathways. We identified the transcripts related to each gene involved in flavonoid and vitamin C biosynthesis. Several *cytochrome P450s* (*CYPs*) and *glucosyltransferases* (*GTs*) genes involved in flavonoid biosynthesis and various other metabolic pathways were also documented. Further, 6510 transcription factors and 4420 EST derived simple sequence repeat (SSR) markers were also predicted. The present study enlightened various characteristic features of *P. emblica* genome, and provided an important resource for future molecular and functional genomics studies.

## Introduction

*Phyllanthus emblica* (syn. *Emblica officinalis*, family Euphorbiaceae, *n* = 49) is a deciduous tree distributed across the subtropical and tropical regions of Asian countries such as India, China, Pakistan, Srilanka, Indonesia etc. It is a rich source of bioactive molecules like ascorbic acid (vitamin C), flavonoids, phenolics, terpenoids, tannins, rutin, curcuminoids, emblicol, phyllembelic acid, phyllembelin, emblicanin A, emblicanin B, ellagitannin, ellagic acid, gallic acid, essential amino acids, and alkaloids (Kumar et al., 2007; Poltanov et al., 2009; Krishnaveni and Mirunalini, 2010). In traditional medicines, its fruit and other parts have been extensively used in various herbal formulations to treat a variety of maladies (Perianayagam et al., 2004; Poltanov et al., 2009). Several studies suggested beneficial effects of *P. emblica* in digestion improvement, hyperthermia, blood pressure normalization, assuages asthma, hair growth, and heart and liver reinforcement. It is also useful in the treatment of various eye ailments, dyspepsia, gastroenteritis, anemia, hyperglycemia, fatigue, and general weakness (Perianayagam et al., 2004; Kumaran and Karunakaran, 2006; Kumar et al., 2007, 2008). The extracts of *P. emblica* possess antimicrobial, antioxidant, anticancer, antigenotoxic, anti-inflammatory, hepatoprotective, hypocholesterolemic, antiviral, and antifungal, hypolipidemic, antimutagenic, and immunomodulatory activities (Kumaran and Karunakaran, 2006; Kumar et al., 2007; Chatterjee et al., 2011; Singh et al., 2013). The phenolic compounds especially flavonoids in combination with vitamin C are the major secondary metabolites present in *P. emblica*.

The flavonoids are diverse class of secondary metabolites that have pivotal role in plant growth, development and defense mechanism (Dixon and Steele, 1999; Winkel-Shirley, 2001). They play critical role in the production of plant pigments and involved in various other activities including UV protection and pathogen defense, along with their nutraceutical value in the human diet (Winkel-Shirley, 2001). The structural and regulatory genes involved in flavonoid biosynthesis have been extensively characterized in various plants for their spatial and temporal regulation (Boss et al., 1996; Ban et al., 2007; Singh et al., 2008; Niu et al., 2010).

Plants are rich source of water soluble vitamin C, which plays diverse role in various biological functions in plants and humans as well. In plants, it is involved in biosynthesis of ethylene, gibberellins, and plant pigments, cell growth regulation, acts as an enzyme cofactor in photosynthesis and various other vital functions (Smirnoff and Wheeler, 2000). Further, it is essential in ameliorating the harmful effects of reactive oxygen species derived from chloroplast in photosynthetic eukaryotes (Wheeler et al., 1998). The human beings are largely dependent upon plants for their regular uptake of vitamin C due to the lack of an enzyme gulonolactone oxidase involved in final step of vitamin C biosynthesis. It acts as a cofactor for enzymes involved in the post-translational hydroxylation of collagen, carnitine biosynthesis, involved in conversion of the neurotransmitter dopamine to norepinephrine, and in tyrosine metabolism (Diliberto and Daniels, 1991). It also plays vital role in regulation of iron uptake, cardiovascular functions, maintenance of cartilage, and wound healing.

The high throughput transcriptome sequencing and analyses have become a versatile method for gene discovery and expression profiling in recent years (Kalra et al., 2013; Chen et al., 2015). The Illumina sequencing technology has proven to be an exceptionally successful in a wide variety of whole-transcriptome investigations, particularly for the characterization of non-model organisms where reference genome is not available (Wilhelm et al., 2008; Tang et al., 2011; Chen et al., 2015; Kumar S. et al., 2015). Several computational tools for *de novo* assembly of short read sequence data and identification of genes involved in various metabolic pathways have also been demonstrated (Pertea et al., 2003; Zerbino and Birney, 2008; Grabherr et al., 2011; Fu et al., 2012).

Molecular insights into the medicinal plants have gained attention in recent years. The availability of genomic and transcriptomic data of such plants has been comprehensively reviewed by Misra (2014). Despite of high medicinal value, the genomic information of *P. emblica* is still very limited. To the best of our knowledge, only 71 ESTs were available in the National Center for Biotechnology Information (NCBI) database before the start of this work. The inadequate genomic/transcriptomic data was a major bottleneck in understanding various molecular mechanisms and biosynthetic pathways including flavonoids and vitamin C biosynthesis in *P. emblica*. Earlier, we developed a method for RNA isolation (Kumar and Singh, 2012) and cloned *flavonoid 3 hydroxylase* (*F3H*) gene of flavonoid biosynthetic pathway using primers based PCR approach from this plant (Kumar A. et al., 2015). However, we could not get complete information about biosynthetic pathways and other molecular details using that approach. Therefore, to gain further insight into metabolic and molecular networks of this plant, the *de novo* transcriptome study was initiated with foremost emphasis to investigate the candidate genes involved in flavonoids and vitamin C biosynthesis.

## Materials and methods

### Plant material, RNA isolation, and transcriptome sequencing

Young leaves from the top aerial part of tree at the edge of branchlets (Supplementary Figure S1) and full bloom flowers were harvested from approximately 10-year-old healthy plant of *P. emblica* growing under natural environmental conditions in the botanical garden of the Panjab University, Chandigarh, India. Samples were harvested in early morning of November month, snap frozen in liquid nitrogen, and stored at −80°C till further use. Total RNA was isolated using the method described by Kumar and Singh (2012), followed by RNA purification and on column DNase I digestion using miRNA Easy kit (Qiagen, Germany). The cDNA library was prepared using TruSeq™ RNA Sample preparation kit (Illumina, USA) at Microarray core facility, Huntsman Cancer Institute, University of Utah, Salt Lake City, Utah, USA, followed by 50 cycled single end library sequencing on Illumina Hiseq 2000 sequencing platform.

### *De novo* assembly and sequence clustering

Computational analysis was carried out on HP workstation with eight cores, 2.27 GHz Intel Xeon processor with 16 GB RAM. Data was filtered to remove adapter sequences by using the fastx_clipper tool of the FASTX Toolkit (www.hannonlab.cshl.edu/fastxtoolkit) with exact matching of target sequence. Reads passing phred quality scores ≥20 (an error probability of 0.01) were filtered out, and unambiguous sequences (“N”) were trimmed. The *de novo* assembly of filtered reads was performed using a short read assembler program, VELVET (Version 0.7.55) (Zerbino and Birney, 2008) followed by OASES program (Version 0.1.11) (Schulz et al., 2012) with different k-mer hash length.

After assembly, the clustering tool CD-HIT-EST was used to cluster nearly identical (>99%) transcripts. The longest sequence within each cluster was extracted. The clustering process was supplemented with TGICL-CAP3 clustering program and the clustered contigs and singletons were merged to get final transcript assembly. Statistical parameters such as total transcripts, average size of transcript, transcripts having length ≥1000 bp etc. were used to assess assembly quality. In order to assess the reliability of assembly, assembled sequences were further validated using previously characterized *P. emblica* gene sequences available at NCBI Genbank database. BLASTN analysis was performed for each reported sequence against the set of assembled sequences at *e*-value 10^−5^.

### Sequence annotation and classification

The contigs and singletons were annotated using FastAnnotator (bioinfo.cgu.edu.tw/fastannotator_release). It utilizes Blast2GO, PRIAM, and RPS-BLAST to assign Gene ontology (GO) term, enzyme commission (EC) codes and protein domains. BLASTX was run against NCBI non-redundant (NR) protein database. The query sequences were assigned with a cut-off e-value of 10^−5^. Assembled transcripts were also searched against plant transcription factor database (PlnTFDB; http://plntfdb.bio.uni-potsdam.de/v3.0/downloads.php) for the identification of transcription factor families. Genes involved in flavonoid and vitamin C biosynthesis were sorted out and analyzed by BLASTX analysis.

### Simple sequence repeat (SSR) identification

To assess SSR markers present in the transcriptome of *P. emblica*, Microsatelite searching tool (MISA, http://pgrc.ipk-gatersleben.de/misa/) was used to mine potential microsatellites in the assembled unigenes. The mono-nucleotide (10 times), di-nucleotides (4 times), tri-, tetra-, penta-, and hexanucleotide (3 times) were searched.

## Results and discussion

### Transcriptome sequencing and *de novo* assembly

The high-throughput transcriptome sequencing of *P. emblica* using Illumina Hiseq 2000 (Illumina, USA) produced 32,382,864 single end reads. Since the 3′ end of reads are more prone to error, two bases from 3′ end were excluded after quality assessment. The 48 bases high quality reads were used for further analysis. After adapter trimming and removal of un-ambiguous sequences, 31,285,965 reads were assembled into 134,205 unique sequences and 89,242 singletons (Table 1). The *de novo* assembly was optimized at different k-mer lengths, in which k-mer 33 was found best with 358 bp N50 value. The longest and average contig length was 5418 and 278 bp, respectively (Table 1). Although, 100–199 bp contigs were abundant, yet 11,074 contigs had sequence length ≥500 bp. Contigs with ≤ 100 bp length were discarded from further analysis. The accuracy of assembled sequences was validated by aligning them with the ESTs of *P. emblica* available at NCBI database. Significant hits were observed with ESTs with an average 82.72% similarity.

**Table 1:** **Summary of transcriptome data generated on Illumina Hiseq2000 for ***P. emblica*****.

Total number of single-end reads	323,828,64
Number of reads obtained after quality filtering	31,285,965
Number of assembled transcripts	91,288
Singletons	89,242
Longest contig length	5418
Average length of transcripts (bp)	278
Total base covered (bp)	25,400,212

### Sequence clustering and similarity search

**Figure 1:**
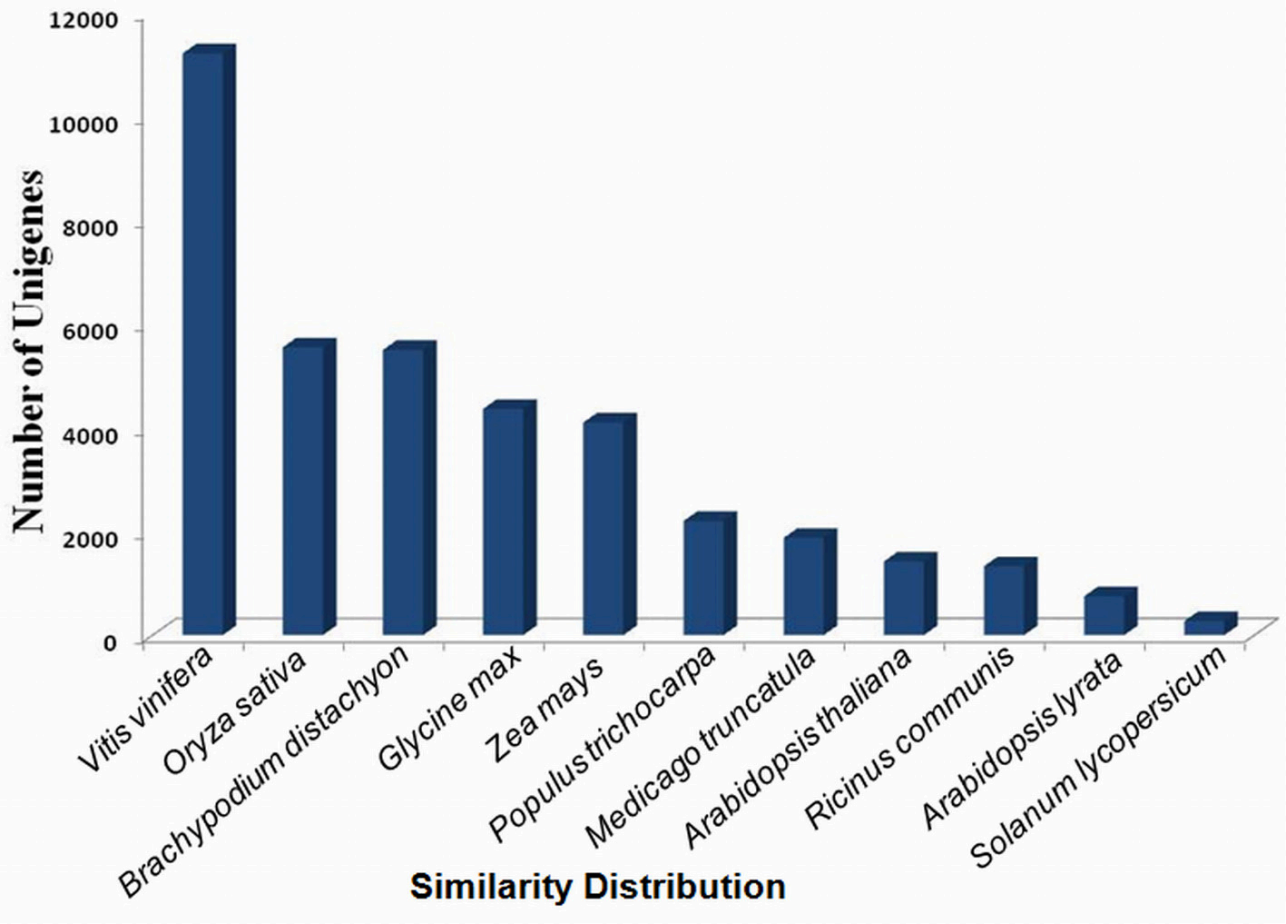
**Similarity distribution with different plant species using the NR protein database (with an ***e*** ≤ 1e^**−5**^)**.

The assembled sequences were further clustered by hierarchical clustering and re-assembled using Contig Assembly Program (CAP3). This step reduced the total number of uniquely assembled contigs from 134,205 to 91,288 (Supplementary Table S1 and Supplementary Figure S2). BLAST search using Fastannotator at *e* ≤ 1e^−5^ showed significant similarity of 47,267 (51.7%) sequences with the NCBI-NR protein database. The e-value distribution analysis showed that 18.52% sequences matched with e < 1e^−50^, whereas 81.48% with *e*-value ranged between 1e^−5^ and 1e^−50^ (Supplementary Figure S3). The conserved domain database search for protein functional domains indicated protein kinases, leucine rich repeats, WD40, CAZ-associated structural protein, RNA recognition motifs, mothers against decapentaplegic, pentatricho peptide repeats, zinc finger-C3HC4, and CYP450 were highly represented domains (Supplementary Figure S4). About 77% sequences showed >60% similarity with the matched sequences in the database (Supplementary Figure S5). In terms of similarity with other species, 29% transcripts showed high similarity with the genes of *Vitis vinifera*, followed by *Oryza sativa* “japonica group” (14.35%), *Brachipodium distachyon* (14.23%), *Glycine max* (11.28%), *Zea mays* (10.60%), *Populus trichocarpa* (5.68%), *Medicago truncatula* (4.84%), and *Ricinus communis* (3.43%) (Figure 1).

### Functional annotation and classification

**Figure 2:**
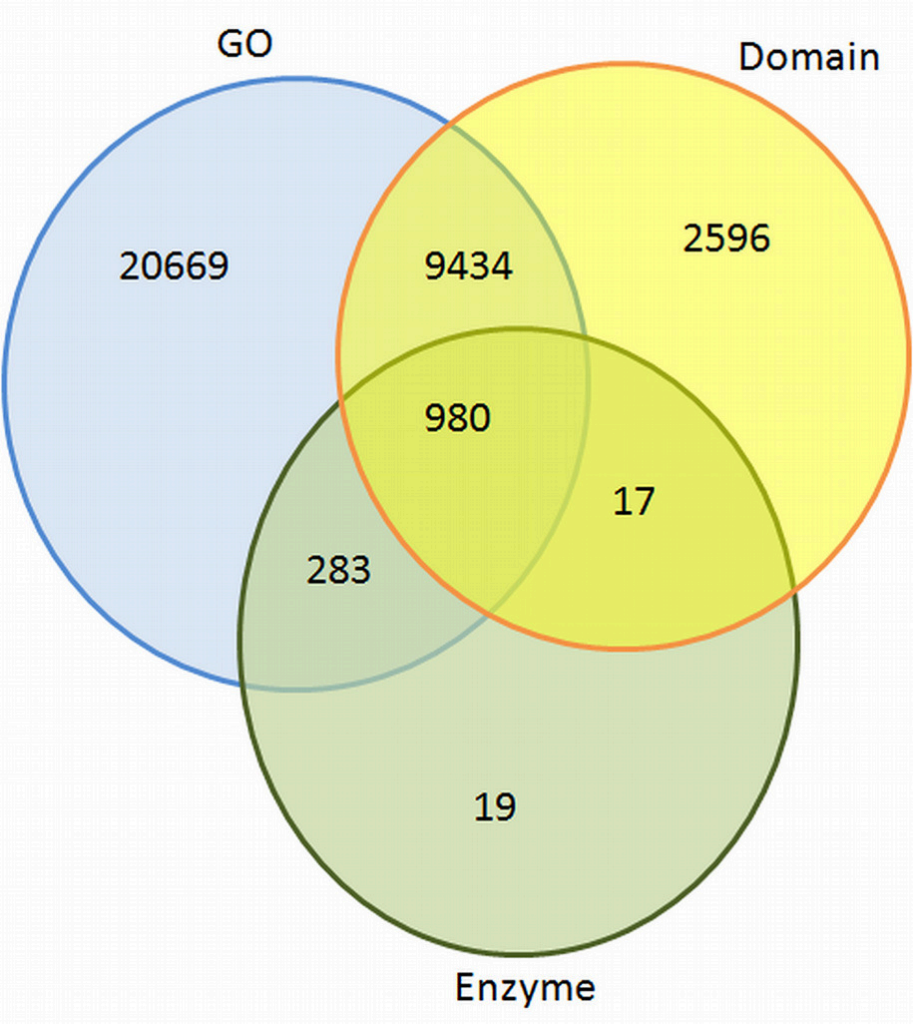
**Overview of similarities of contigs found against GO, domain, and EC databases**.

It was crucial to gather in-depth functional information to understand various metabolic processes. Therefore, the transcripts sequences were used for BLAST search (*e*-value 1e^−05^) against gene ontology (GO) and enzyme classification (EC) databases (Supplementary Table S2). An overview of annotated transcripts against GO, EC, and protein domains databases is shown in Figure 2.

### GO annotation

**Figure 3:**
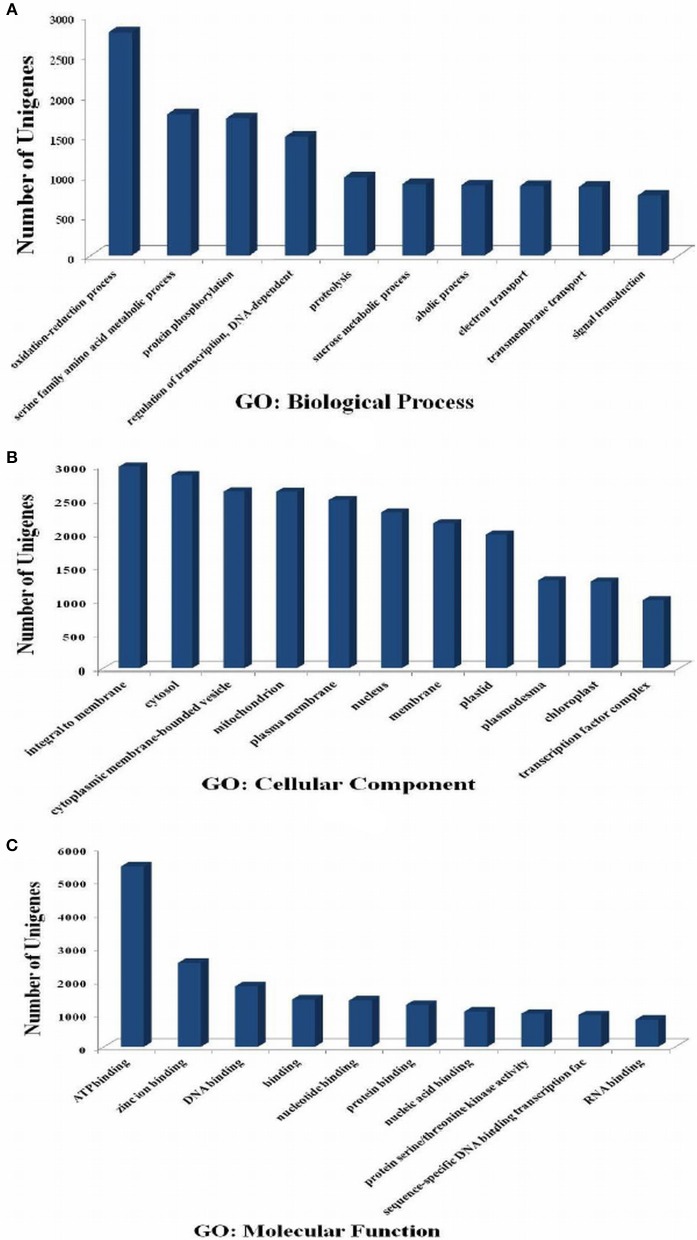
**Gene Ontology (GO) classification of the ***P. emblica*** transcriptome**. GO term are summarized into three main categories (**A**-biological process, **B**-cellular component, and **C**-molecular function) based on significant hits of unigenes against the NR database.

GO analysis provides functional classification of gene, which defines the properties of genes and their products. GO has three ontologies; molecular function, cellular components, and biological processes. A total of 31,366 transcripts were annotated using GO database. In biological process category of GO (Figure 3A), oxidation-reduction process (GO: 0055114), serine family amino acid metabolic process (GO: 0009069), protein phosphorylation (GO: 0006468), regulation of transcription (GO: 0006355), and proteolysis (GO:0006508) were highly represented GO categories. Under cellular component, sequences related to the integral to membrane (GO:0016021), cytosol (GO:0005829), and cytoplasmic membrane-bounded vesicle (GO:0016023) were most enriched categories (Figure 3B). However, ATP binding (GO:0005524), DNA binding (GO:0003677), protein binding (GO:0005515), and protein serine/threonine kinase activity (GO:0004674) were highly enriched in molecular function category (Figure 3C).

### EC classification

**Figure 4:**
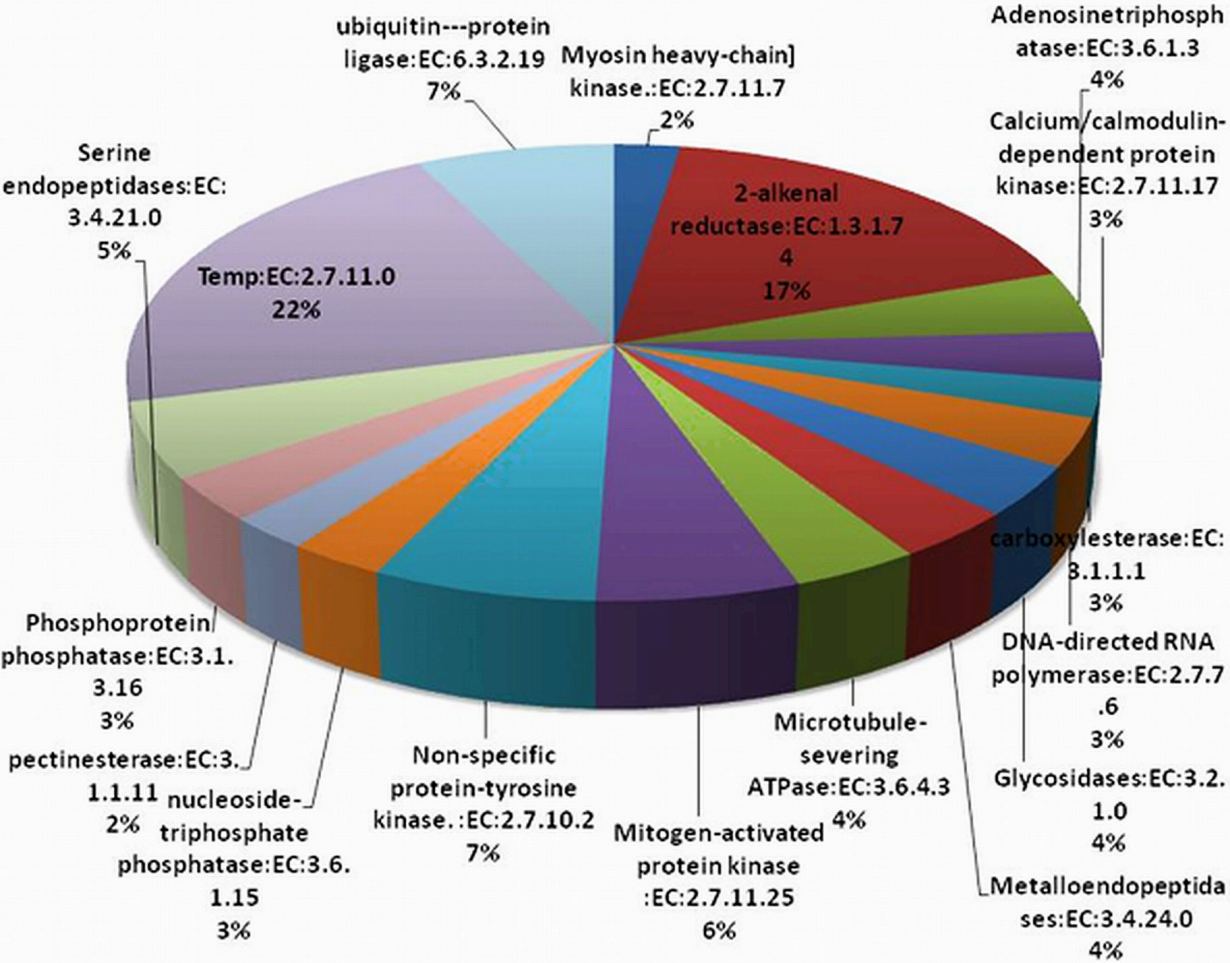
**Abundance of enzyme classes (Top 20) in ***P. emblica*** transcriptome**. Area under each pie represents the % value of actual number of transcripts.

The EC annotation was obtained for 1299 transcript sequences. Top 20 abundant enzymes predicted in *P. emblica* transcriptome are shown in Figure 4. Non-specific serine/threonine protein kinase enzyme family (368 transcripts) members were present in high numbers. Due to the lack of tyrosine kinase receptors, tyrosine phosphorylation is less common in plants as compared to serine-threonine kinases (Mano et al., 2005). Although a number of dual—specificity kinases have been found in plants systems, but none of the true protein-tyrosine kinases (PTK) have been reported, except two PTKs being predicted in *A. thaliana* (Rudrabhatla et al., 2006; Miranda-Saavedra and Barton, 2007). Around 17% of transcripts were characterized as 2-alkenal reductase (AER, EC 1.3.1.7.4). AER plays central role in the detoxification of reactive carbonyls. The reduction of α, β unsaturated bonds present in reactive carbonyls is carried out by AER and are involved in anti-oxidative defense in plants (Luan, 2002). AER plays an important role in oxidation-reduction processes, amino acid transport, and response to various stress conditions, hence the putative role of AER in *P. emblica* could be response to oxidative stress. About 7% transcripts of *P. emblica* represented Ubiquitin-protein ligase (EC 6.3.2.19). They are involved in the regulation of various metabolic processes e.g., vegetative growth control mediated by hormones, plant reproduction, stress tolerance, and DNA repair (Mazzucotelli et al., 2006). E3 ubiquitin-ligases are also known to regulate signaling pathways initiated by ABA induced stress (Lyzenga et al., 2011).

### Analysis of the transcripts encoding transcription factors (TFs)

**Figure 5:**
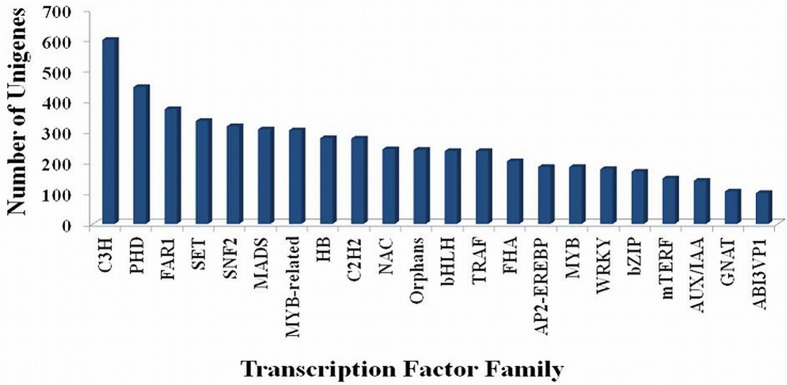
**Number of unigenes of ***P. emblica*** matching with different transcription factor families**. Top 22 families are shown here and rest are mentioned in Supplementary Table S3.

Transcripts encoding TFs were identified by sequence comparison to known TF gene families. In total, 6510 (7.10%) putative transcripts showing similarity to TF genes were identified in *P. emblica* (Supplementary Table S3). These included TF families like C3H, PHD, FAR1, MADS, SET, SNF2, MYB, bHLH etc (Figure 5). Out of them, C3H (601 transcripts), PHD (447 transcripts), and FAR1 (375 transcripts) were most abundant, and GNAT and ABI3VP1 were least represented. Several C3H proteins have been reported to participate in developmental responses and hormonal pathways. Thus, C3H is expected to play significant role in stress responses and various metabolic processes in *P. emblica*, also.

The flavonoid pathway genes are mainly regulated at transcription level (Winkel-Shirley, 2001). Several TFs regulating flavonoid biosynthesis have been identified in plants (Davies and Schwinn, 2003; Allan et al., 2008; Palapol et al., 2009; Niu et al., 2010). The R2R3-MYB and bHLH TFs form the MBW complex with the WD40 proteins, which may regulate the transcription of various genes of flavonoid biosynthetic pathway in plants. This regulation is via specific binding to motifs in the promoter region (Hernandez et al., 2004; Hartmann et al., 2005; Dare et al., 2008). The basic helix loop-helix (bHLH) family of TFs is one of the most abundant and highly conserved in plant kingdoms. These bind the E-box (CANNTG), although most plant bHLHs specifically recognize the so-called G-box (CACGTTG). The bHLH proteins are known to regulate biological processes i.e., light signaling, hormone signaling, wound and drought stress response, organ, and tissue development. The R2R3-MYB and bHLH TFs responsible for the anthocyanin accumulation have been well characterized (Ban et al., 2007; Espley et al., 2007). In grapes *R2R3-MYB* TF, *VvMYBA* activates the *UDP-glucose:flavonoid-3-O-glycosyltranferase* (*UFGT*) gene, which plays a key role in color development (white and red) in grape skin (Boss et al., 1996; Kobayashi et al., 2002). *PpMYB10* of peach bind to *dihydroflavonol 4-reductase* (*DFR)* promoter and activated the anthocyanin biosynthesis in tobacco and *Arabidopsis* (Lin-Wang et al., 2010).

In *Arabidopsis*, three closely related *MYBs, AtMYB11, AtMYB12*, and *AtMYB111* regulate *AtFLS1* and other steps for production of flavonol glucosides (Mehrtens et al., 2005; Stracke et al., 2007). These genes share significant similarity and form subgroup 7 of *R2R3-MYB* gene family. Due to the functional similarity amongst *MYB11, MYB12*, and *MYB111*, they show target specificity for flavonoid biosynthetic pathway genes such as *CHS, CHI, F3H*, and *FLS1*. The *FLS* is regulated by light and UV exposure via activation of *MYB* TFs in grapes and maize, respectively (Czemmel et al., 2009; Ferreyra et al., 2012). At least four *MYB* TFs (*VvMYB5a, VvMYB5b, VvMYBPA1*, and *VvMYBPA2*) are reported in grapes that regulate key steps of the flavonoid pathway. They affect accumulation of proanthocyanidins in leaves, flowers and in early berry development, before the véraison stage (Terrier et al., 2009).

### Analysis of metabolic pathway genes

#### Flavonoid biosynthesis pathway

**Figure 6:**
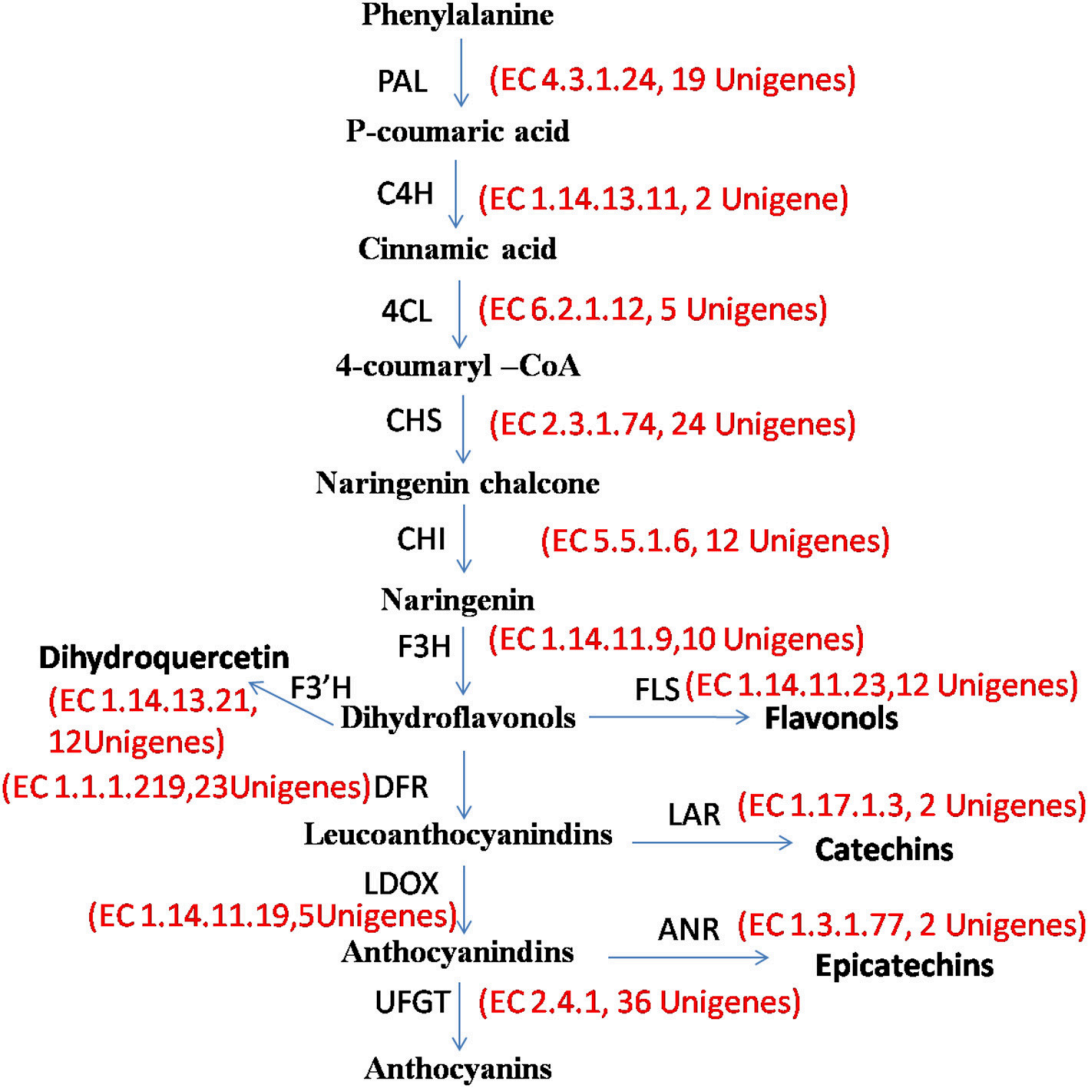
*****P. emblica*** unigenes involved in flavonoid biosynthesis pathway**. Number in brackets following EC number indicates the number of unigenes identified for the corresponding gene.

All the genes associated with flavonoid biosynthesis pathway were detected in the transcriptome data of *P. emblica* (Figure 6) with multiple transcripts for each gene (Supplementary Table S4). Flavonoids are synthesized via the phenylpropanoid pathway, wherein phenylalanine gets deaminated to form cinnamic acid by phenylalanine ammonia lyase (EC 4.3.1.24, 19 transcripts). The hydroxylation of cinnamic acid by cinnamate 4-hydroxylase (EC 1.14.13.11, 2 transcripts) produces *p*-coumaric acid, which subsequently converted to *p*-coumaroyl-CoA by 4-coumaroyl CoA ligase (EC 6.2.1.12, 5 unigenes). Further, Chalcone synthase (EC 2.3.1.74, 24 unigenes) catalyses chalcone production by condensation of 4-coumaroyl-Co A and malonyl-Co A in 1:3 ratio. After two-steps of condensation catalyzed by chalcone synthase and chalcone isomerase (EC 5.5.1.6, 12 unigenes), a flavanone called naringenin is produced. It acts as a precursor of many flavonoid and isoflavonoid compounds. The naringenin is oxidized by flavanone 3 hydroxylase (EC 1.14.11.9, 12 unigenes) into dihydroflavonols. The hydroxylation of naringenin by flavonoid 3′-hydroxylase (EC 1.14.13.21, 9 unigenes) and flavonoid 3′, 5′-hydroxylase (EC 1.14.13.88, 0 unigene) yields eriodictyol and dihydrotricetin, respectively. Naringenin, eriodictyol, and dihydrotricetin are flavanones which are involved in plant stress responses. Flavone synthase (EC 1.14.11.22, 1 unigene) catalyzes the conversion of flavanones to flavones, and flavanone 3-hydroxylase (EC 1.14.11.9, 10 unigenes) can convert these flavanones to dihydroflavonols. The dihydroflavonols lead to the production of flavonols and flavan- 3, 4-diols (leucoanthocyanidin), by the actions of flavonol synthase (EC 1.14.11.23, 12 unigenes) and dihydroflavonol 4-reductase, a NADPH-dependent enzyme (EC 1.1.1.219, 23 unigenes), respectively. Leucoanthocyanidins are converted either to anthocyanidins through the action of anthocyanidin synthase (EC 1.14.11.19, 5 unigene) or reduced to catechins by anthocyanidin reductase (EC 1.3.1.77, 2 unigenes). Catechins are bioprospecting flavonoids as they have huge manifestation in human health and are also involved in plant growth and survival. Hence, they might be potential targets for metabolic engineering (Rani et al., 2012).

These annotations were useful in predicting the molecular functions of unigenes and constructing the metabolic pathways in *P. emblica*. The knowledge of flavonoid biosynthetic pathways along with the regulatory TFs (such as *MYB, bHLH*, and *WD40*-type) rendered metabolic engineering much simplified for the production of various essential metabolites.

Isoflavonoids are an important subclass of flavonoids being involved in plant defense and nodulation (Dixon and Steele, 1999). Certain transcripts associated with isoflavonoid metabolism such as *isoflavone 7-O-methyltransferase, 2-hydroxy isoflavanone synthase, isoflavone 2*′*-hydroxylase, isoflavone 4*′*-O-methyltransferase*, and *isoflavone reductase* were also present in our data.

### Discovery of transcripts encoding for *CYPs* and *GTs*

CYP450s form the largest superfamily of enzymes because of its prevalence in plant systems and implications in plant metabolism. The role of CYP450s in catalyzing a wide range of regio-specific, stereospecific and irreversible steps involved in plant metabolite biosynthesis is well documented (Renault et al., 2014). A total of 214 unique transcripts were annotated as CYP450s (Supplementary Table S5). Amongst all, *cinammate 4-hydroxylase* (2 unigenes), *flavanone 3-hydroxylase* (EC 1.14.11.9, 12 unigenes), *flavone synthase II* (EC 1.14.11.22, 3 unigene), and *flavonoid 3*′*-hydroxylase* (EC 1.14.13.21, 9 unigenes) are known to be involved in flavonoid biosynthesis.

The P450 monooxygenases are heme protein—dependent mixed-function oxidase systems. They utilize NADPH/NADH to reduce atmospheric dioxygen and yield an organic substrate along with a water molecule. They are involved in several processes such as hydroxylation, dealkylation, dimerizations, epoxidation, isomerization, deamination etc. Schuler and Werck-Reichhart (2003).

Activities of P450s are categorized into two classes- first exists in biosynthetic pathways and second in detoxification pathways (Schuler, 1996; Chapple, 1998). They play important role during synthesis of lignin, flavonoids, coumarins, sinapoyl esters, isoflavonoids, hydroxamic acids, glucosinolates, terpenes, gibberellins, brassinosteroids, auxin, and oxygenated fatty acids. In addition to these known biosynthetic activities, plant P450s are also responsible for catabolizing a range of endogenous and toxic exogenous compounds encountered in the environment such as herbicides, insecticides, and some pollutants (Werck-Reichhart et al., 2000; Harvey et al., 2002).

Several unigenes encoding different types of glycosyltransferases were also found in our dataset (Supplementary Table S6). Out of them, UFGTs supposed to be involved in flavonoid biosynthesis included *flavonoid glucosyl-transferase, UDP-glucose:isoflavone 7-O-glucosyltransferase, tetrahydroxychalcone glucosyltransferase*, and *anthocyanidin 3-O-glucosyltransferase*.

The glycosylation of anthocyanidin resulted into increase in stability, decrease in reactivity, and change in its spectral features; otherwise anthocyanidins are highly reactive and inherently unstable. Major increase in anthocyanin accumulation is attributed to higher mRNA levels of *UFGT, CHS*, and *F3H* genes (Prior and Wu, 2006; Petrussa et al., 2013). *UFGT* is the final gene in anthocyanin pathway and play vital role in anthocyanin biosynthesis and accumulation. The UFGT mediated transfer of the glucosyl moiety from UDP-glucose to hydroxyl groups of anthocyanidins is shown crucial for their stability and solubility. The expression of *UFGT* is reported to be controlled by various transcription factors like *MYB10, MYB123*, and *bHLH3* (Ravaglia et al., 2013).

### Vitamin C (L-Ascorbic Acid) biosynthesis

**Figure 7:**
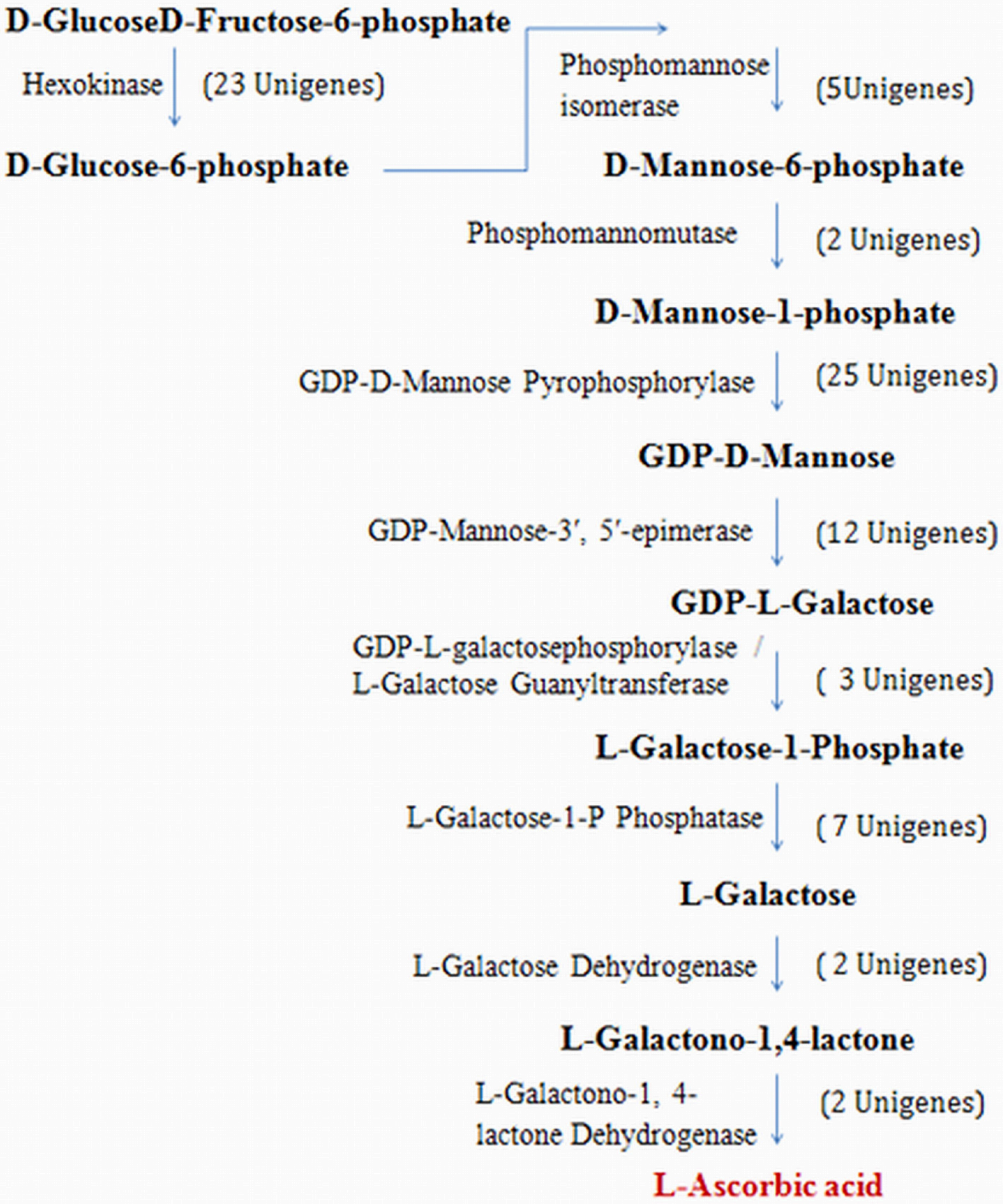
*****P. emblica*** unigenes involved in LAA (vitamin C) biosynthetic pathway**. Number in brackets indicates the number of unigenes identified for the corresponding gene.

*P. emblica* contains high levels of vitamin C, therefore transcriptome analysis is an indispensable tool to investigate the genes involved in its biosynthesis. Transcripts related to each gene involved in biosynthesis of L-Ascorbic Acid (AsA) were identified in the current study (Figure 7). The number of contigs matching with the pathway genes varies from 2 for *L-galactose dehydrogenase* to more than 20 for *Hexokinase* and *GDP-D-Mannose pyrophosphorylase* (Supplementary Table S7). AsA biosynthesis in plants occurs through various routes but activation of each pathway is dependent upon species and developmental stages of the plant. The first proposed pathway for AsA biosynthesis proceeds through GDP-D-mannose and L-galactose (Wheeler et al., 1998). In 1963, it was demonstrated in strawberry fruits, where 6-carbon labeled molecule D-glucose converted into AsA without cleavage of the carbon chain. Later on, it was postulated that L-galactono-1, 4-lactone is the immediate precursor for AsA.

D-Glucose is the main precursor for ascorbic acid biosynthesis, which gets converted into D-glucose 6-phosphate by the enzyme hexokinase (EC 2.7.1.1), a component of glycolysis. The glucose ring is phosphorylated by addition of a phosphate group derived from ATP. D-Glucose-6-phosphate isomerized into D-fructose-6-Phosphate with the action of phosphoglucose isomerase (EC 5.3.1.9, 364 unigenes) by rearrangements of carbon oxygen bond to transform 6-C ring into 5-C ring. It further converted mannose-6-phosphate by phosphomannose isomerase (EC 5.3.1.8, 5 unigenes). Subsequently phosphomannomutase (EC 5.4.2.8, 3 unigenes) transfers the phosphate group from 6-carbon position to 1-carbon position to produce D-mannose-1-phosphate. Then, GDP-D-mannose pyrophosphorylase (EC 2.7.7.22, 19 unigenes) adds one glycosyl unit to produce GDP-D-mannose, which epimerizes at 3′ and 5′ positions to yield GDP-L-galactose by enzyme GDP-mannose-3′, 5′-epimerase (EC 5.1.3.18, 23 unigenes). GDP-mannose pyrophosphorylase is reported to have significant role in regulation of AsA biosynthesis. In fact, a correlation between mRNA levels of *GDP-mannose pyrophosphorylase* and AsA levels has been documented in many plant species (Badejo et al., 2007, 2009).

The conversion of GDP-L-galactose into L-galactose-1-phosphate is catalyzed by GDP-L-galactose phosphorylase (EC 2.7.7.69, 14 unigenes). L-galactose-1-phosphate is regarded as the first metabolite dedicated to AsA biosynthesis. Hence, this step represents the first committed step in the whole pathway. Further, L-galactose-1-phosphate leads to the formation of L-galactose by the activity of L-galactose-1-P phosphatase (EC 3.1.3.25, 14 unigenes). L-galactono-1, 4-lactone is produced by the activity of L-galactose dehydrogenase (EC 1.1.1.316, 14 unigenes) from L-galactose. It is postulated that regulation of L-galactose dehydrogenase expression is light dependent and light-dependent changes in respiration might directly affect their activity (Tamaoki et al., 2003; Bartoli et al., 2006). Finally, L-ascorbic acid is produced from L-galactose by the action of L-galactono-1,4-lactone dehydrogenase (EC 1.3.2.3, 5 unigenes) enzyme, which is highly specific for L-galactono-1, 4-lactone.

The enzyme GDP-mannose 3′, 5′-epimerase is responsible for the catalytic conversion of GDP-D-mannose into GDP-L-galactose. A novel compound, GDP-L-gulose is also produced by the 5′-epimerization of GDP-D-mannose. Therefore, it was postulated that the GDP-mannose-3′, 5′-epimerase enzyme catalyses distinct epimerization reactions depending upon the molecular form of enzyme leading to the formation of either GDP-L-galactose or GDP-L-gulose (Wolucka and Van Montagu, 2003). Here, we found a new branch point of this pathway in plants and a connecting link with the pathway operating in animals. Cats and dogs can synthesize their own vitamin C unlike humans, because human cells cannot perform the conversion of 1-gulono-1,4-lactone into ascorbic acid, which is catalyzed by the enzyme gulonolactone oxidase. It was observed that the gene *gulonolactone oxidase* is present in humans, but it is a non-functional pseudogene because of accumulation of several mutations over the time (De Tullio, 2010).

### SSR analysis

A total of 4420 SSRs were identified in 4079 transcripts of *P. emblica*, in which 314 sequences contained more than 1 SSR (Table 2). With a frequency of over 43.5% (1925/4420), di-nucleotides were most abundant SSRs, which was followed by tri-nucleotides (33.2%, 1469/4420), tetra-nucleotides (0.045%, 20/4420) and penta-nucleotides (0.013%, 6/4420). The results indicated that transcripts containing SSR markers were indeed abundant in *P. emblica*. In particular, dinucleotide SSRs were identified within the sequences of flavonoid biosynthetic pathway genes such as *PAL, CHI*, and *DFR*. In vitamin C biosynthesis pathway, SSR motifs were identified in four genes (Table 3). Additionally, di-nucleotide to tetra-nucleotide repeats were predicted in various other genes. Therefore, in future, research on various isoforms and activities of genes involved in flavonoid and vitamin C biosynthesis may be helpful in explaining, why *P. emblica* has such a high amount of flavonoids and vitamin C?

**Table 2:** **Simple sequence repeats (SSRs) identified in transcripts of ***P. emblica*****.

**SSR mining**	**Number**
Total number of sequences examined:	91,288
Total size of examined sequences (Mb):	25.42
Total number of identified SSRs:	4420
Number of SSR containing sequences:	4079
Number of sequences containing more than one SSR:	314
Number of SSRs present in compound formation:	248
**DISTRIBUTION OF SSRs IN DIFFERENT REPEAT TYPES**	
**Unit size**	**Number of SSRs (%)**
Mononucleotide	986
Dinucleotide	1925
Trinucleotide	1479
Tetranucleotide	20
Pentanucleotide	6
Hexanucleotide	14

**Table 3:** **Simple sequence repeats (SSRs) identified in the genes involved in flavonoid and vitamin C biosynthesis**.

**Gene name**	**Contig ID**	**SSR motif**
**FLAVONOID BIOSYNTHESIS**		
*Phenylalanine ammonia lyase*	NODE_135356	(A)_11_
*Chalcone isomerase*	NODE_45303	(GA)_20_
*Dihydroflavonol reductase*	NODE_6237	(CT)_22_
**VITAMIN C BIOSYNTHESIS**		
*Phosphoglucoseisomerase*	NODE_119675	(GA)_5_
*GDP-D-Mannose pyrophosphorylase*	NODE_25565	(GCAT)_3_
*L-Galactose-1-P phosphatase*	NODE_15063	(TG)_5_
*L-Galactose dehydrogenase*	NODE_14146	(GGC)_4_

## Data archiving statement

Transcriptome data of this study can be accessed at NCBI SRA database under SRA ID SRP075209 (Bioproject PRJNA313483).

## Author contributions

Conceived and designed the experiments: KS Performed the experiments: AK, SK, SB Analyzed the data: SK, SB, VV, RK Contributed reagents/materials/analysis tools: KS, JK, RK, SK Wrote the paper: KS, RK, BS.

### Conflict of interest statement

The authors declare that the research was conducted in the absence of any commercial or financial relationships that could be construed as a potential conflict of interest.

## Supplementary material

Supplementary Figure S1 **Herbarium record of the ***P. emblica*** plant deposited in the herbarium of Botany department of Panjab University, Chandigarh, India (recford no 21074)**. Position of young leaves have been marked.

Supplementary Figure S2 **Size distribution of the contigs obtained from ***de novo*** assembly of high quality clean reads**.

Supplementary Figure S3 **E-value distribution of the BLAST hits for unigenes of ***P. emblica*** (E-value cut off 1e^**−5**^)**.

Supplementary Figure S4 **Conserved domains distribution of the of the best BLAST hits for each unigene**.

Supplementary Figure S5 **Similarity distribution of the best BLAST hits for unigenes**.

Supplementary Table S1 **Sequences of assembled transcripts of ***P. emblica*****.

Supplementary Table S2 **Annotation of ***P. emblica*** transcriptome against NR protein, GO and other databases by BLAST analysis**.

Supplementary Table S3 **Details on all the transcription factor (TF) families analyzed in ***P. emblica*****.

Supplementary Table S4 **Details on all the genes of flavonoid biosynthesis pathway analyzed in ***P. emblica*** transcriptome**.

Supplementary Table S5 **Annotated cytochrome P450s (CYPs) in ***P. emblica*** transcriptome**.

Supplementary Table S6 **Annotated glycosyltransferases (GTs) in ***P. Emblica*** transcriptome**.

Supplementary Table S7 **Details of all the genes involved in vitamin C biosynthesis identified in ***P. emblica*** transcriptome**.
