# Supplementary figures and images for: De novo Transcriptome Analysis Revealed Genes Involved in Flavonoid and Vitamin C Biosynthesis in Phyllanthus emblica (L.)
Source: Front Plant Sci. 2016 Oct 27;7:1610. doi: 10.3389/fpls.2016.01610 (PMC5081490; doi:10.3389/fpls.2016.01610)

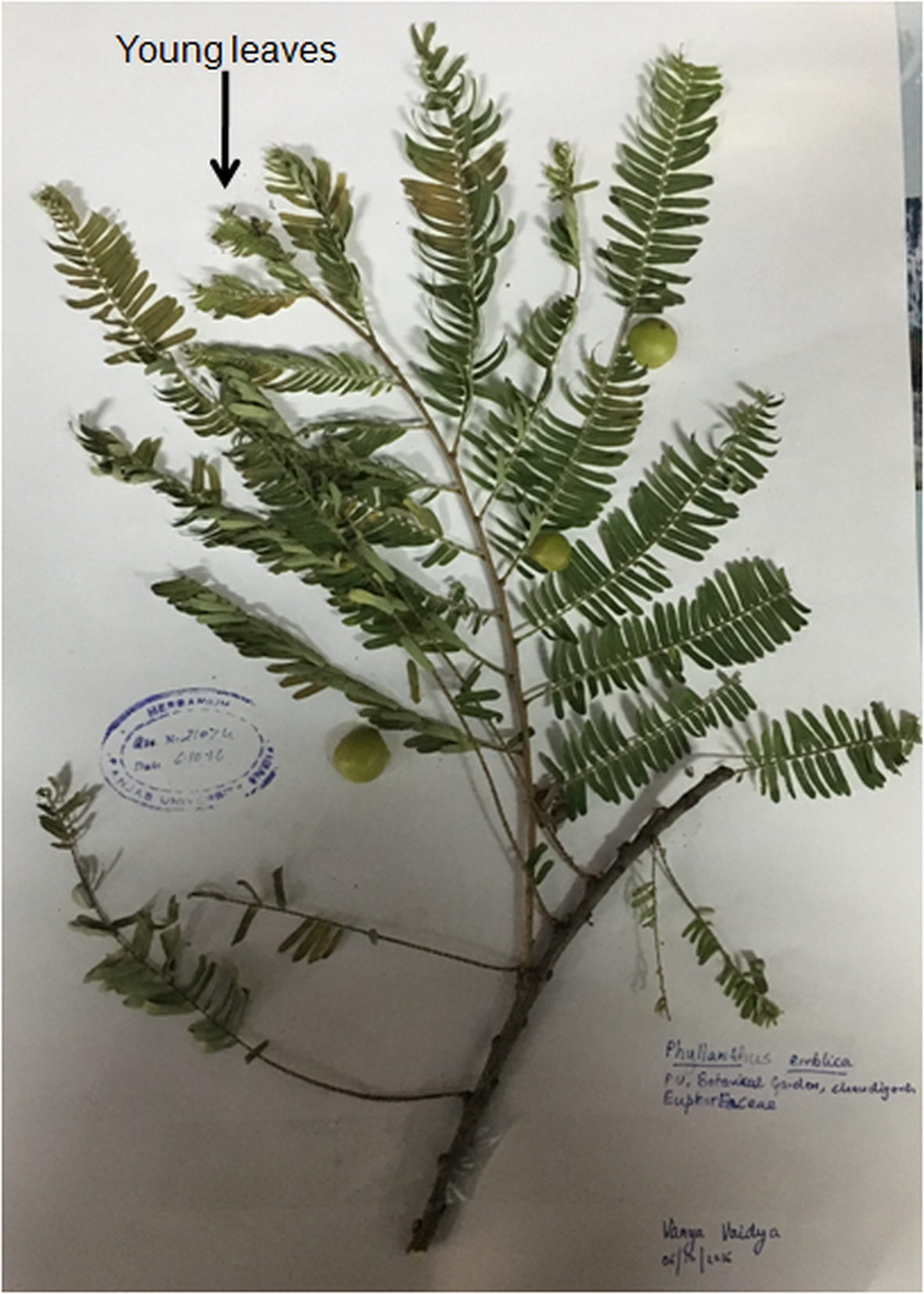

Supplement: Supplementary Figure S1 — Herbarium record of the P. emblica plant deposited in the herbarium of Botany department of Panjab University, Chandigarh, India (recford no 21074). Position of young leaves have been marked. [file Image1.TIF]

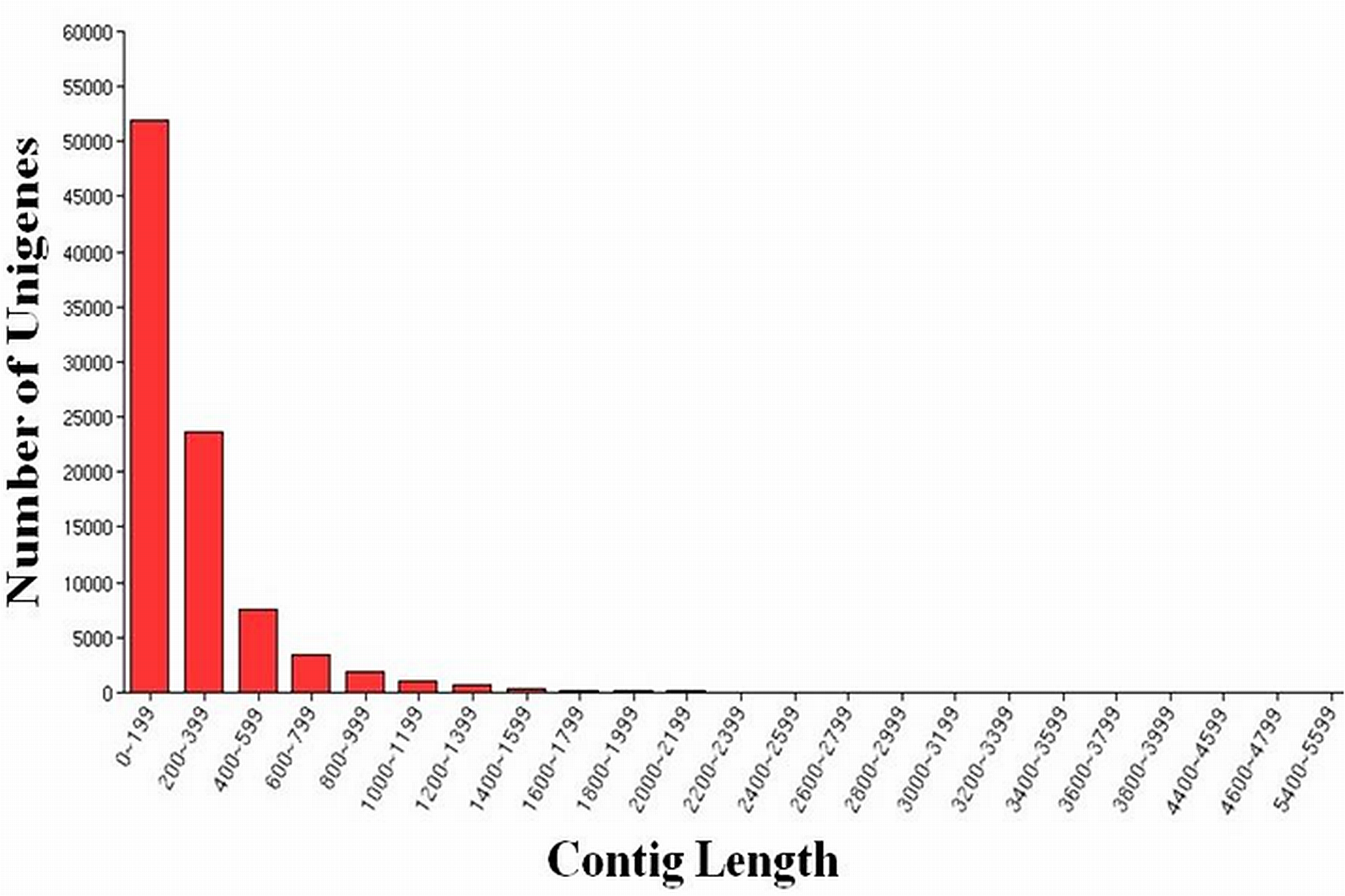

Supplement: Supplementary Figure S2 — Size distribution of the contigs obtained from de novo assembly of high quality clean reads. [file Image2.TIF]

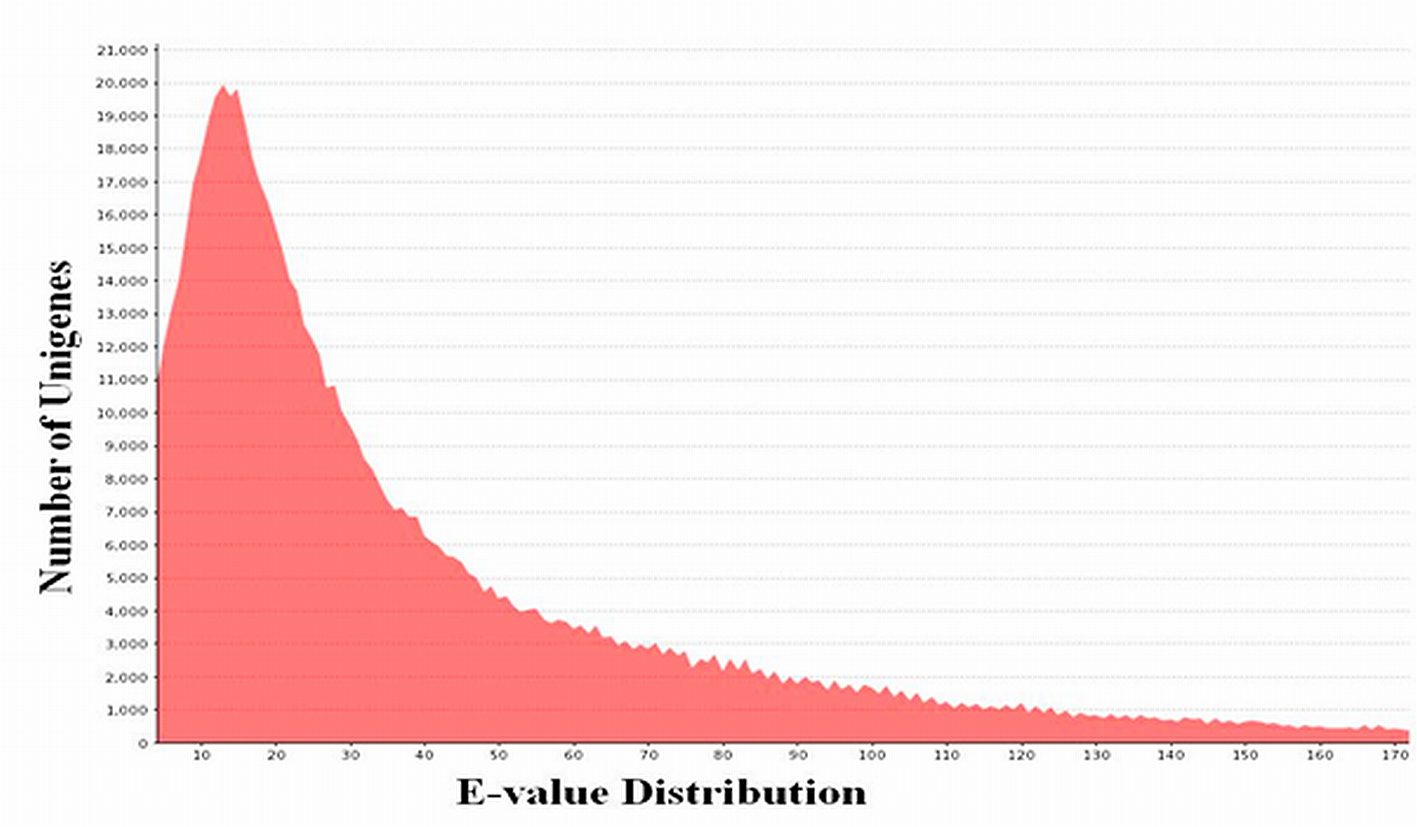

Supplement: Supplementary Figure S3 — E-value distribution of the BLAST hits for unigenes of P. emblica (E-value cut off 1e−5). [file Image3.TIF]

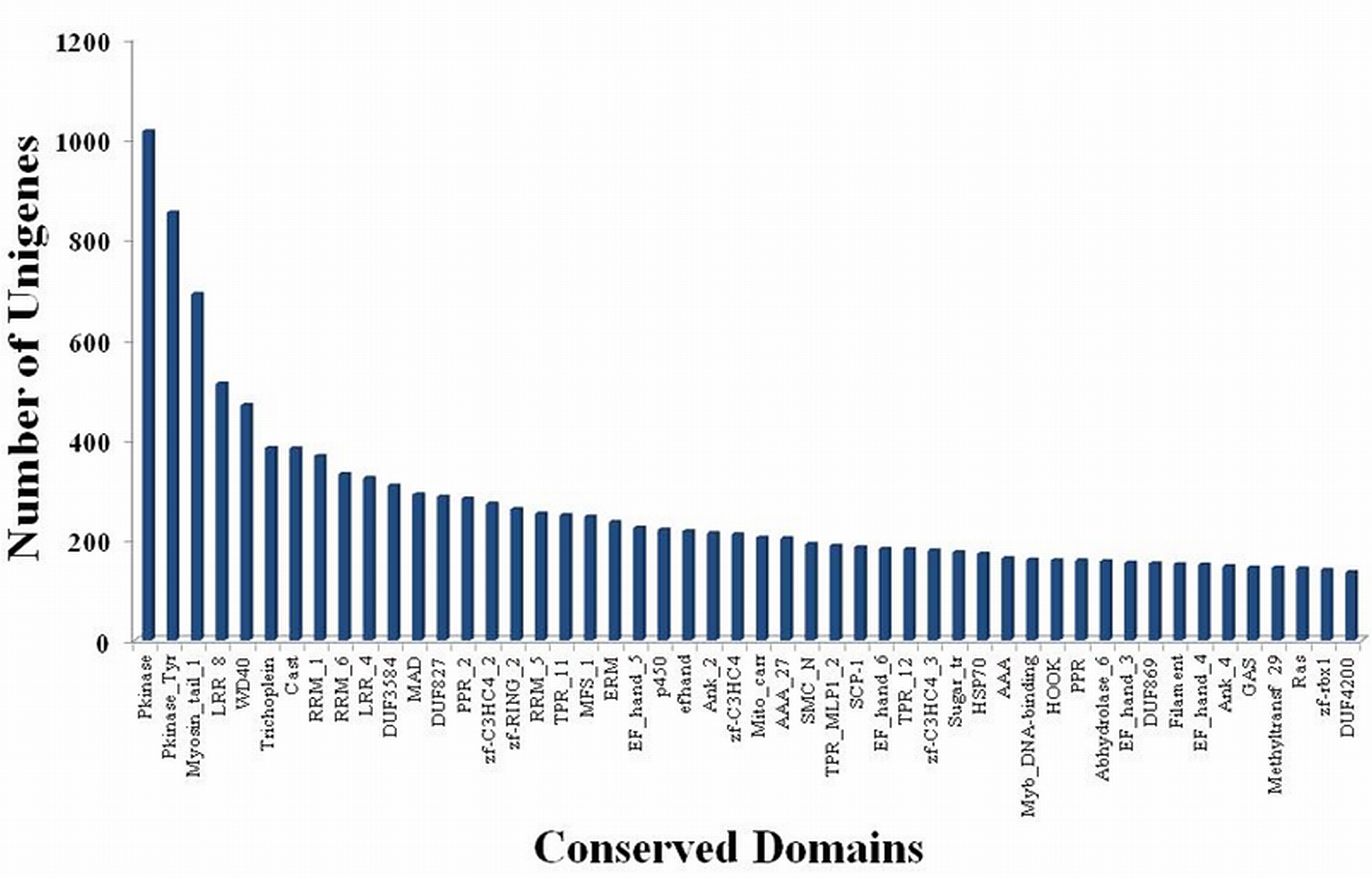

Supplement: Supplementary Figure S4 — Conserved domains distribution of the of the best BLAST hits for each unigene. [file Image4.TIF]

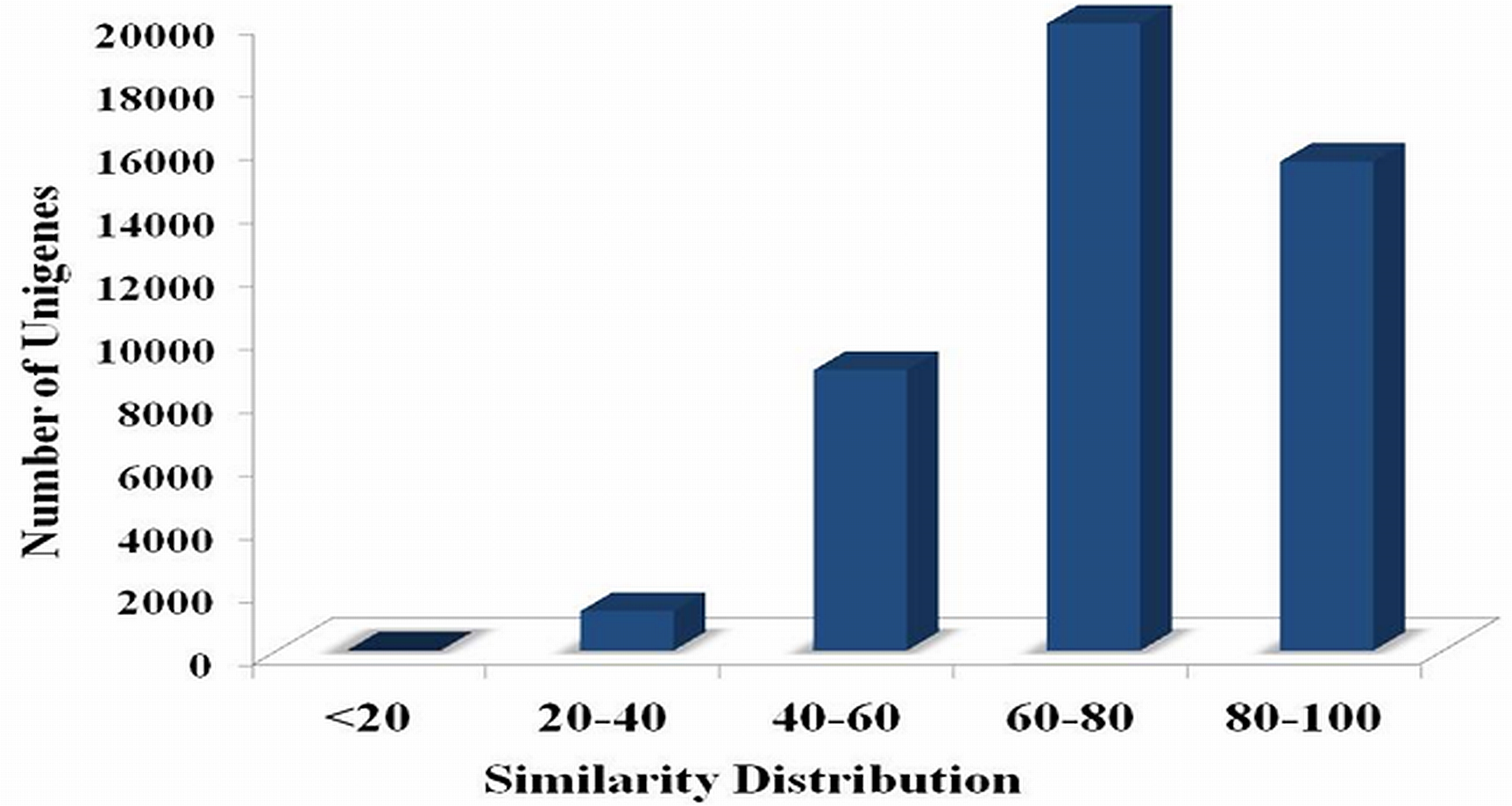

Supplement: Supplementary Figure S5 — Similarity distribution of the best BLAST hits for unigenes. [file Image5.TIF]
