# Supplementary material for: De novo Transcriptome Analysis Revealed Genes Involved in Flavonoid and Vitamin C Biosynthesis in Phyllanthus emblica (L.)
Source: Front Plant Sci. 2016 Oct 27;7:1610. doi: 10.3389/fpls.2016.01610 (PMC5081490; doi:10.3389/fpls.2016.01610)
Supplement: Supplementary Table S3 — Details on all the transcription factor (TF) families analyzed in P. emblica. [file Table3.DOC]

**Supplementary table S3: Details on all the transcription factor (TF) families analyzed in *P. emblica***

| **Transcription factor family Name** | **Number** |
| --- | --- |
| C3H | 601 |
| PHD | 447 |
| FAR1 | 375 |
| SET | 336 |
| SNF2 | 319 |
| MADS | 309 |
| MYB-related | 306 |
| HB | 280 |
| C2H2 | 279 |
| NAC | 244 |
| Orphans | 242 |
| bHLH | 238 |
| TRAF | 238 |
| FHA | 205 |
| AP2-EREBP | 186 |
| MYB | 186 |
| WRKY | 179 |
| bZIP | 171 |
| mTERF | 149 |
| AUX/IAA | 141 |
| GNAT | 106 |
| ABI3VP1 | 101 |
| SBP | 91 |
| Jumonji | 87 |
| G2-like | 85 |
| GRAS | 75 |
| DBP | 65 |
| C2C2-GATA | 64 |
| Trihelix | 63 |
| ARF | 62 |
| CCAAT | 57 |
| LUG | 52 |
| Tify | 48 |
| DDT | 47 |
| TCP | 45 |
| TUB | 45 |
| C2C2-CO-like | 41 |
| BSD | 40 |
| HSF | 37 |
| BES1 | 35 |
| SWI/SNF-BAF60b | 35 |
| HMG | 31 |
| CAMTA | 30 |
| ARID | 29 |
| LOB | 28 |
| CPP | 26 |
| EIL | 26 |
| SWI/SNF-SWI3 | 24 |
| C2C2-Dof | 23 |
| TAZ | 23 |
| E2F-DP | 21 |
| OFP | 21 |
| PLATZ | 21 |
| RWP-RK | 21 |
| LIM | 20 |
| ARR-B | 19 |
| CSD | 19 |
| GRF | 17 |
| Alfin-like | 16 |
| Sigma70-like | 16 |
| RB | 15 |
| PBF-2-like | 14 |
| BBR/BPC | 13 |
| Pseudo | 13 |
| C2C2-YABBY | 11 |
| HRT | 11 |
| TIG | 11 |
| zf-HD | 10 |
| Coactivator | 8 |
| GeBP | 8 |
| Rcd1-like | 7 |
| IWS1 | 4 |
| MBF1 | 4 |
| S1Fa-like | 3 |
| ULT | 2 |
| VOZ | 2 |
| LFY | 1 |
| MED6 | 1 |
| MED7 | 1 |
| SOH1 | 1 |
| SRS | 1 |
