# Supplementary material for: De novo Transcriptome Analysis Revealed Genes Involved in Flavonoid and Vitamin C Biosynthesis in Phyllanthus emblica (L.)
Source: Front Plant Sci. 2016 Oct 27;7:1610. doi: 10.3389/fpls.2016.01610 (PMC5081490; doi:10.3389/fpls.2016.01610)
Supplement: Supplementary Table S4 — Details on all the genes of flavonoid biosynthesis pathway analyzed in P. emblica transcriptome. [file Table4.DOC]

**Supplementary table S4: Details on all the genes of flavonoid biosynthesis pathway analyzed in *P. emblica* transcriptome.**

| **CONTIG ID** | **LENGTH (bp)** | **BLAST HIT** | **E-VALUE** | **GENE** | **PLANT** |
| --- | --- | --- | --- | --- | --- |
| NODE_122333 | 433 | tr|Q84TM3|Q84TM3_ALLCE | 2.00E-55 | *Chalcone synthase B* | *Allium cepa (Onion)* |
| NODE_134083 | 460 | tr|Q84TM3|Q84TM3_ALLCE | 4.00E-42 | *Chalcone synthase B* | *A. cepa* |
| NODE_156026 | 584 | tr|Q84TM3|Q84TM3_ALLCE | 1.00E-96 | *Chalcone synthase B* | *A. cepa* |
| NODE_657 | 129 | tr|Q1W9N0|Q1W9N0_ANTAD | 2.00E-15 | *Chalcone synthase* | *Anthurium andraeanum* |
| NODE_134081 | 769 | tr|Q84L24|Q84L24_ANTAD | 1.00E-83 | *Putative chalcone synthase* | *A. andraeanum* |
| NODE_172630 | 112 | tr|F4K3J3|F4K3J3_ARATH | 9.00E-09 | *Chalcone synthase* | *Arabis purpurea* |
| NODE_9993 | 146 | tr|I1IMN5|I1IMN5_BRADI | 2.00E-09 | *Chalcone synthase* | *Brassica rapa var. purpuraria* |
| NODE_183495 | 463 | tr|A8Q8J7|A8Q8J7_BRUMA | 2.00E-08 | *Chalcone synthase 2* | *Camellia chekiangoleosa* |
| NODE_25910 | 1396 | tr|A8PZN1|A8PZN1_BRUMA | 2.00E-12 | *Chalcone synthase 2* | *C. chekiangoleosa* |
| NODE_18992 | 320 | tr|H6UJ36|H6UJ36_CAMSI | 2.00E-58 | *Chalcone synthase 1* | *Caragana arborescens (Siberian pea tree)* |
| NODE_120988 | 177 | tr|A6MZ92|A6MZ92_GLYUR | 3.00E-28 | *Chalcone synthase* | *Gossypium hirsutum (Upland cotton) (G. mexicanum)* |
| NODE_179969 | 124 | tr|Q677B3|Q677B3_HYAOR | 4.00E-12 | *Chalcone synthase* | *Hydrangea macrophylla* |
| NODE_150770 | 611 | tr|Q5YJM1|Q5YJM1_HYAOR | 4.00E-58 | *Chalcone synthase* | *Ipomoea batatas (Sweet potato) (Convolvulus batatas)* |
| NODE_656 | 140 | tr|Q9ZQS6|Q9ZQS6_IPOBA | 5.00E-17 | *Chalcone synthase* | *Lilium hybrid* |
| NODE_4513 | 105 | tr|G7IN58|G7IN58_MEDTR | 1.00E-08 | *Chalcone synthase* | *Mimulus aurantiacus* |
| NODE_123093 | 1115 | tr|C0LZ70|C0LZ70_NANDO | 3.00E-75 | *Chalcone synthase* | *Narcissus tazetta var. chinensis* |
| NODE_69872 | 139 | tr|C0LZ70|C0LZ70_NANDO | 3.00E-15 | *Chalcone synthase* | *N. tazetta var. chinensis* |
| NODE_107217 | 292 | tr|C7EYJ4|C7EYJ4_NARPS | 3.00E-48 | *Chalcone synthase* | *N. tazetta var. chinensis* |
| NODE_3044 | 119 | tr|G1FET0|G1FET0_PAELC | 1.00E-05 | *Chalcone synthase (Naregenin-chalcone synthase)* | *Persea americana (Avocado)* |
| NODE_97001 | 314 | tr|O82416|O82416_PAPSO | 2.00E-39 | *Chalcone synthase (Naringenin-chalcone synthase J)* | *Petunia hybrida* |
| NODE_127772 | 282 | tr|Q5ZF91|Q5ZF91_PLAMJ | 3.00E-34 | *Chalcone synthase* | *Populus alba (White poplar)* |
| NODE_29638 | 201 | tr|B9RUA7|B9RUA7_RICCO | 7.00E-20 | *Chalcone synthase 3 (Naringenin-chalcone synthase 3)* | *Ruta graveolens (Common rue)* |
| NODE_33546 | 1154 | tr|B9SN62|B9SN62_RICCO | 7.00E-76 | *Chalcone synthase 3 (Naringenin-chalcone synthase 3)* | *R. graveolens (Common rue)* |
| NODE_55588 | 190 | sp|Q7XA39|RGA4_SOLBU | 3.00E-19 | *Chalcone synthase 1* | *Solanum lycopersicum (Tomato) (Lycopersicon esculentum)* |
| NODE_132630 | 144 | tr|M8AUT1|M8AUT1_AEGTA | 9.00E-12 | *Dihydroflavonol-4-reductase* | *Aegilops tauschii (Tausch's goatgrass) (A. squarrosa)* |
| NODE_163177 | 158 | tr|M8CBI3|M8CBI3_AEGTA | 5.00E-13 | *Dihydroflavonol-4-reductase* | *A. tauschii (Tausch's goatgrass) (A. squarrosa)* |
| NODE_46174 | 208 | tr|H6X1M6|H6X1M6_9ROSI | 2.00E-13 | *Dihydroflavonol-4-reductase* | *Ectocarpus siliculosus (Brown alga)* |
| NODE_145057 | 229 | tr|Q5W957|Q5W957_9LILI | 1.00E-12 | *Dihydroflavonol-4-reductase* | *E. siliculosus (Brown alga)* |
| NODE_135568 | 176 | tr|G7JIM0|G7JIM0_MEDTR | 1.00E-19 | *Dihydroflavonol-4-reductase* | *Medicago truncatula (Barrel medic) (M. tribuloides)* |
| NODE_90964 | 124 | tr|G7KUL5|G7KUL5_MEDTR | 3.00E-06 | *Dihydroflavonol-4-reductase* | *M. truncatula (Barrel medic) (M. tribuloides)* |
| NODE_160294 | 1139 | tr|G7I4V2|G7I4V2_MEDTR | 1.00E-58 | *Dihydroflavonol-4-reductase* | *M. truncatula (Barrel medic) (M. tribuloides)* |
| NODE_185289 | 119 | tr|E1U253|E1U253_WHEAT | 3.00E-08 | *Dihydroflavonol-4-reductase* | *Triticum urartu (Red wild einkorn) (Crithodium urartu)* |
| NODE_8435 | 548 | tr|E1U253|E1U253_WHEAT | 7.00E-16 | *Dihydroflavonol-4-reductase* | *T. urartu (Red wild einkorn) (Crithodium urartu)* |
| NODE_30738 | 380 | tr|F6LAX4|F6LAX4_WHEAT | 6.00E-62 | *Dihydroflavonol-4-reductase* | *T. urartu (Red wild einkorn) (Crithodium urartu)* |
| NODE_22139 | 167 | tr|B6TNE5|B6TNE5_MAIZE | 6.00E-24 | *Dihydroflavonol-4-reductase* | *Zea mays* |
| NODE_104704 | 119 | tr|B6TU35|B6TU35_MAIZE | 4.00E-10 | *Dihydroflavonol-4-reductase* | *Z. mays* |
| NODE_20211 | 850 | tr|B6SRS7|B6SRS7_MAIZE | 8.00E-81 | *Dihydroflavonol-4-reductase* | *Z. mays* |
| NODE_20212 | 904 | tr|B6SRS7|B6SRS7_MAIZE | 1.00E-79 | *Dihydroflavonol-4-reductase* | *Z. mays* |
| NODE_127619 | 167 | sp|Q6QHK0|CFI_ALLCE | 2.00E-18 | *Chalcone--flavonone isomerase (Chalcone isomerase)* | *A. cepa* |
| NODE_17902 | 146 | sp|Q6QHK0|CFI_ALLCE | 4.00E-14 | *Chalcone--flavonone isomerase (Chalcone isomerase)* | *A. cepa* |
| NODE_45303 | 744 | sp|Q6QHK0|CFI_ALLCE | 2.00E-27 | *Chalcone--flavonone isomerase (Chalcone isomerase)* | *A. cepa* |
| NODE_103129 | 153 | tr|D5YEG5|D5YEG5_MYCTU | 1.00E-06 | *Chalcone isomerase* | *N. tazetta var. chinensis* |
| NODE_182944 | 161 | tr|G0YGJ7|G0YGJ7_MYTED | 1.00E-15 | *Chalcone isomerase* | *N. tazetta var. chinensis* |
| NODE_1173 | 386 | tr|C0LZ70|C0LZ70_NANDO | 2.00E-23 | *Chalcone isomerase* | *N. tazetta var. chinensis* |
| NODE_1181 | 262 | tr|C0LZ70|C0LZ70_NANDO | 1.00E-21 | *Chalcone isomerase* | *N. tazetta var. chinensis* |
| NODE_133156 | 759 | tr|Q84TM2|Q84TM2_ALLCE | 3.00E-128 | *Flavanone 3-hydroxylase* | *A. cepa* |
| NODE_61314 | 177 | tr|F4JWP9|F4JWP9_ARATH | 3.00E-19 | *Flavanone 3-hydroxylase-like protein* | *Arabidopsis thaliana (Mouse-ear cress)* |
| NODE_144497 | 1323 | tr|Q93XV7|Q93XV7_BRUGY | 0 | *Flavanone 3-hydroxylase* | *C. sinensis (Tea)* |
| NODE_49380 | 760 | tr|D5LMH3|D5LMH3_LILLO | 7.00E-128 | *Flavanone 3-hydroxylase* | *Litchi chinensis (Lychee)* |
| NODE_87484 | 169 | tr|Q8W314|Q8W314_ORYSJ | 4.00E-24 | *Putative flavanone 3-hydroxylase* | *Oryza sativa subsp. japonica (Rice)* |
| NODE_71286 | 156 | tr|Q6ZKY1|Q6ZKY1_ORYSJ | 2.00E-09 | *Flavanone 3-hydroxylase* | *Parrya nudicaulis* |
| NODE_112836 | 99 | tr|B9HHA9|B9HHA9_POPTR | 7.00E-09 | *Flavanone 3-hydroxylase* | *P. trichocarpa (Western balsam poplar) (P. balsamifera subsp. trichocarpa)* |
| NODE_12215 | 237 | tr|D5MAF8|D5MAF8_TRIRP | 3.00E-30 | *Flavanone 3-hydroxylase* | *T. aestivum (Wheat)* |
| NODE_151748 | 127 | tr|Q6QHK3|Q6QHK3_ALLCE | 4.00E-15 | *Phenylalanine ammonia-lyase* | *A. cepa* |
| NODE_135366 | 371 | tr|Q8H2A5|Q8H2A5_ANACO | 3.00E-55 | *Phenylalanine ammonia-lyase* | *Ananas comosus (Pineapple) (A. ananas)* |
| NODE_171043 | 223 | tr|Q3SAX1|Q3SAX1_GOSHI | 1.00E-36 | *Phenylalanine ammonia-lyase* | *Helianthus annuus (Common sunflower)* |
| NODE_51188 | 611 | tr|D6MKG9|D6MKG9_9ASPA | 4.00E-90 | *Phenylalanine ammonia-lyase* | *Lycoris radiata (Hurricane lily) (Red spiderlily)* |
| NODE_5140 | 533 | tr|D6MKL9|D6MKL9_9ASPA | 2.00E-66 | *Phenylalanine ammonia-lyase* | *L. radiata* |
| NODE_78326 | 124 | tr|Q8RVK4|Q8RVK4_MUSAC | 4.00E-15 | *Phenylalanine ammonia-lyase* | *Musa acuminata (Banana) (M. cavendishii)* |
| NODE_78333 | 170 | tr|Q8RVK4|Q8RVK4_MUSAC | 1.00E-23 | *Phenylalanine ammonia-lyase* | *M. acuminata* |
| NODE_90386 | 532 | tr|Q8RVK4|Q8RVK4_MUSAC | 4.00E-63 | *Phenylalanine ammonia-lyase* | *M. acuminata (* |
| NODE_59752 | 211 | tr|Q1EPA8|Q1EPA8_MUSAC | 2.00E-18 | *Phenylalanine ammonia-lyase* | *M. acuminata AAA Group (dessert banana)* |
| NODE_24037 | 938 | tr|H2E607|H2E607_MUSAC | 6.00E-157 | *Phenylalanine ammonia-lyase* | *M. balbisiana (Banana)* |
| NODE_155300 | 905 | tr|Q06AC3|Q06AC3_MUSAC | 6.00E-51 | *Phenylalanine ammonia-lyase* | *M. balbisiana* |
| NODE_169035 | 262 | tr|A1EGX7|A1EGX7_MUSAC | 1.00E-22 | *Phenylalanine ammonia-lyase* | *M. balbisiana* |
| NODE_109787 | 230 | tr|C7EYJ4|C7EYJ4_NARPS | 2.00E-37 | *Phenylalanine ammonia-lyase* | *N. tazetta var. chinensis* |
| NODE_64354 | 385 | tr|Q9AWU5|Q9AWU5_ORYSJ | 1.00E-49 | *Phenylalanine ammonia-lyase* | *O. sativa subsp. japonica* |
| NODE_122660 | 146 | tr|Q42425|Q42425_PANMI | 2.00E-18 | *Phenylalanine ammonia-lyase* | *P. americana (Avocado)* |
| NODE_57335 | 575 | tr|F2XQG6|F2XQG6_PANVG | 1.00E-39 | *Phenylalanine ammonia-lyase* | *P. americana (Avocado)* |
| NODE_5610 | 454 | tr|B9SFK0|B9SFK0_RICCO | 3.00E-51 | *Phenylalanine ammonia-lyase* | *Ricinus communis (Castor bean)* |
| NODE_111470 | 280 | tr|Q7XJ49|Q7XJ49_ALLCE | 3.00E-13 | *Flavonoid glucosyl-transferase* | *A. cepa* |
| NODE_124890 | 271 | tr|Q7XJ50|Q7XJ50_ALLCE | 1.00E-12 | *Flavonoid glucosyl-transferase* | *A. cepa* |
| NODE_135882 | 266 | tr|Q7XJ49|Q7XJ49_ALLCE | 8.00E-23 | *Flavonoid glucosyl-transferase* | *A. cepa* |
| NODE_135884 | 590 | tr|Q7XJ49|Q7XJ49_ALLCE | 3.00E-74 | *Flavonoid glucosyl-transferase* | *A. cepa* |
| NODE_149468 | 370 | tr|Q7XJ49|Q7XJ49_ALLCE | 4.00E-41 | *Flavonoid glucosyl-transferase* | *A. cepa* |
| NODE_164789 | 127 | tr|Q7XJ52|Q7XJ52_ALLCE | 2.00E-16 | *Flavonoid glucosyl-transferase* | *A. cepa* |
| NODE_168947 | 207 | tr|Q7XJ50|Q7XJ50_ALLCE | 9.00E-12 | *Flavonoid glucosyl-transferase* | *A. cepa* |
| NODE_169683 | 107 | tr|Q7XJ49|Q7XJ49_ALLCE | 4.00E-07 | *Flavonoid glucosyl-transferase* | *A. cepa* |
| NODE_169783 | 210 | tr|Q7XJ51|Q7XJ51_ALLCE | 2.00E-13 | *Flavonoid glucosyl-transferase* | *A. cepa* |
| NODE_41143 | 194 | tr|Q7XJ50|Q7XJ50_ALLCE | 9.00E-07 | *Flavonoid glucosyl-transferase* | *A. cepa* |
| NODE_50404 | 593 | tr|Q7XJ50|Q7XJ50_ALLCE | 4.00E-61 | *Flavonoid glucosyl-transferase* | *A. cepa* |
| NODE_51012 | 194 | tr|Q7XJ49|Q7XJ49_ALLCE | 2.00E-13 | *Flavonoid glucosyl-transferase* | *A. cepa* |
| NODE_63722 | 229 | tr|Q7XJ52|Q7XJ52_ALLCE | 3.00E-22 | *Flavonoid glucosyl-transferase* | *A. cepa* |
| NODE_98052 | 112 | tr|Q7XJ49|Q7XJ49_ALLCE | 1.00E-07 | *Flavonoid glucosyl-transferase* | *A. cepa* |
| NODE_98693 | 374 | tr|Q7XJ50|Q7XJ50_ALLCE | 3.00E-32 | *Flavonoid glucosyl-transferase* | *A. cepa* |
| NODE_93652 | 561 | tr|Q4VG19|Q4VG19_LILLO | 4.00E-50 | *UDP-glucose flavonoid glucosyl-transferase* | *Litchi chinensis (Lychee)* |
| NODE_94898 | 325 | tr|Q8W314|Q8W314_ORYSJ | 6.00E-45 | *Putative flavonoid glucosyl-transferase* | *O. sativa subsp. japonica* |
| NODE_22606 | 765 | tr|Q9FLR5|Q9FLR5_ARATH | 4.00E-58 | *UDP-glucosyl transferase 73B2 (Flavonol 7-O-glucosyltransferase) (UDP glucose:flavonoid 7-O-glucosyltransferase)* | *A. thaliana* |
| CL1781Cntig1 | 146 | tr|F4HTY0|F4HTY0_ARATH | 2.00E-21 | *UDP-glycosyltransferase 73C6 (Flavonol-3-O-glycoside-7-O-glucosyltransferase 1) (Zeatin O-glucosyltransferase 2)* | *A. thaliana* |
| NODE_118532 | 207 | tr|F4HTY0|F4HTY0_ARATH | 8.00E-16 | *UDP-glycosyltransferase 78D2 (Anthocyanin 3-O-glucosyltransferase) (Flavonol 3-O-glucosyltransferase (UDP glucose:flavonoid 3-O-glucosyltransferase)* | *A. thaliana* |
| NODE_118538 | 176 | tr|F4HTY0|F4HTY0_ARATH | 2.00E-28 | *UDP-glycosyltransferase 78D2 (Anthocyanin 3-O-glucosyltransferase) (Flavonol 3-O-glucosyltransferase.) (UDP glucose:flavonoid 3-O-glucosyltransferase)* | *A. thaliana* |
| NODE_133594 | 275 | tr|Q677C9|Q677C9_HYAOR | 1.00E-20 | *Cyanidin 3-O-rutinoside 5-O-glucosyltransferase (Ih5GT) (Anthocyanin 5-O-glucosyltransferase) (Cyanidin-3-rhamnosylglucoside 5-O-glucosyltransferase) (Uridine diphosphoglucose-cyanidin 3-rhamnosylglucoside 5-O-glucosyltransferase)* | *Iris hollandica (Dutch iris)* |
| NODE_66458 | 221 | tr|Q2QNV2|Q2QNV2_ORYSJ | 1.00E-26 | *Glucosyl transferase (Os01g0176200 protein) (Putative UDP-glucose: flavonoid 7-O-glucosyltransferase) (cDNA clone:002-108-A07* | *O. sativa subsp. japonica* |
| NODE_34561 | 196 | tr|Q7XUF9|Q7XUF9_ORYSJ | 4.00E-23 | *Os01g0176100 protein (Putative UDP-glucose: flavonoid 7-O-glucosyltransferase) (Uncharacterized protein)* | *O. sativa subsp. japonica* |
| NODE_140624 | 1730 | tr|Q6YNS0|Q6YNS0_PRUAV | 6.00E-11 | *Leucoanthocyanidin reductase 2* | *Pyrus communis (Pear) (P. domestica)* |
| NODE_163981 | 164 | tr|Q8W0Q1|Q8W0Q1_WHEAT | 0 | *Leucoanthocyanidin reductase* | *T. urartu (Red wild einkorn) (Crithodium urartu)* |
| NODE_19177 | 364 | tr|O24249|O24249_PRUAR | 3.00E-11 | *Chalcone reductase* | *Pueraria montana var. lobata (Kudzu vine) (P. lobata)* |
| NODE_135876 | 256 | tr|G3E253|G3E253_SCUBA | 2.00E-60 | *Chalcone reductase* | *Sesbania rostrata* |
| NODE_119552 | 314 | tr|B9S3F7|B9S3F7_RICCO | 9.00E-06 | *Leucoanthocyanidin dioxygenase* | *R. communis* |
| NODE_1239 | 168 | tr|B9RLV0|B9RLV0_RICCO | 7.00E-49 | *Leucoanthocyanidin dioxygenase* | *R. communis* |
| NODE_128380 | 233 | tr|B9S3F7|B9S3F7_RICCO | 5.00E-12 | *Leucoanthocyanidin dioxygenase* | *R. communis* |
| NODE_181316 | 811 | tr|B6T822|B6T822_MAIZE | 3.00E-08 | *Leucoanthocyanidin dioxygenase* | *Z. mays* |
| NODE_127506 | 273 | tr|B6UGG5|B6UGG5_MAIZE | 2.00E-65 | *Leucoanthocyanidin dioxygenase* | *Z. mays* |
| NODE_61970 | 773 | sp|O24367|TUR2_SPIPO | 3.00E-22 | *Anthocyanidin reductase* | *Theobroma cacao (Cacao) (Cocoa)* |
| NODE_135056 | 190 | tr|Q9M7B1|Q9M7B1_9ROSI | 7.00E-118 | *Anthocyanidin reductase* | *Vitis vinifera (Grape)* |
| NODE_74022 | 323 | tr|G1UC42|G1UC42_EUSER | 1.00E-17 | *Anthocyanin 3'-O-beta-glucosyltransferase (3'GT)* | *Gentiana triflora (Clustered gentian)* |
| NODE_64254 | 129 | sp|O24367|TUR2_SPIPO | 1.00E-44 | *Anthocyanidin synthase* | |
| NODE_113382 | 304 | tr|D7A6V9|D7A6V9_STAND | 2.00E-08 | *Anthocyanidin synthase* | |
| NODE_145246 | 191 | tr|G7KY73|G7KY73_MEDTR | 2.00E-18 | *Anthocyanin 3'-O-beta-glucosyltransferase* | *M. truncatula (Barrel medic) (M. tribuloides)* |
| NODE_150010 | 126 | tr|M8CYB6|M8CYB6_AEGTA | 1.00E-16 | *Bifunctional dihydroflavonol 4-reductase/flavanone 4-reductase* | *A. tauschii (Tausch's goatgrass) (A. squarrosa)* |
| NODE_62741 | 368 | tr|A5JTT1|A5JTT1_WHEAT | 4.00E-08 | *Bifunctional dihydroflavonol 4-reductase/flavanone 4-reductase* | *T. urartu (Red wild einkorn) (Crithodium urartu)* |
| NODE_132607 | 155 | tr|A7L5U6|A7L5U6_WHEAT | 1.00E-23 | *Bifunctional dihydroflavonol 4-reductase/flavanone 4-reductase* | *T. urartu* |
| NODE_105516 | 431 | tr|E1U253|E1U253_WHEAT | 4.00E-14 | *Bifunctional dihydroflavonol 4-reductase/flavanone 4-reductase* | *T. urartu* |
| NODE_117239 | 2109 | tr|E1U253|E1U253_WHEAT | 2.00E-08 | *Bifunctional dihydroflavonol 4-reductase/flavanone 4-reductase* | *T. urartu* |
| NODE_132028 | 153 | tr|E1U253|E1U253_WHEAT | 0 | *Bifunctional dihydroflavonol 4-reductase/flavanone 4-reductase* | *T. urartu* |
| NODE_103129 | 161 | tr|D5YEG5|D5YEG5_MYCTU | 1.00E-06 | *Chalcone isomerase* | *N. tazetta var. chinensis* |
| NODE_182944 | 386 | tr|G0YGJ7|G0YGJ7_MYTED | 1.00E-15 | *Chalcone isomerase* | *N. tazetta var. chinensis* |
| NODE_1173 | 262 | tr|C0LZ70|C0LZ70_NANDO | 2.00E-23 | *Chalcone isomerase* | *N. tazetta var. chinensis* |
| NODE_1181 | 164 | tr|C0LZ70|C0LZ70_NANDO | 1.00E-21 | *Chalcone isomerase* | *N. tazetta var. chinensis* |
| NODE_19177 | 364 | tr|O24249|O24249_PRUAR | 3.00E-11 | *Chalcone reductase* | *P. montana var. lobata (Kudzu vine) (P. lobata)* |
| NODE_135876 | 129 | tr|G3E253|G3E253_SCUBA | 2.00E-60 | *Chalcone reductase* | *S. rostrata* |
| NODE_657 | 112 | tr|Q1W9N0|Q1W9N0_ANTAD | 2.00E-15 | *Chalcone synthase* | *A. andraeanum (Flamingo lily)* |
| NODE_172630 | 177 | tr|F4K3J3|F4K3J3_ARATH | 9.00E-09 | *Chalcone synthase* | *A. purpurea* |
| NODE_120988 | 124 | tr|A6MZ92|A6MZ92_GLYUR | 3.00E-28 | *Chalcone synthase* | *G. hirsutum (* |
| NODE_179969 | 611 | tr|Q677B3|Q677B3_HYAOR | 4.00E-12 | *Chalcone synthase* | *H. macrophylla (Bigleaf hydrangea)* |
| NODE_150770 | 1115 | tr|Q5YJM1|Q5YJM1_HYAOR | 4.00E-58 | *Chalcone synthase* | *I. batatas (Sweet potato) (Convolvulus batatas)* |
| NODE_123093 | 139 | tr|C0LZ70|C0LZ70_NANDO | 3.00E-75 | *Chalcone synthase* | *N. tazetta var. chinensis* |
| NODE_69872 | 292 | tr|C0LZ70|C0LZ70_NANDO | 3.00E-15 | *Chalcone synthase* | *N. tazetta var. chinensis* |
| NODE_107217 | 282 | tr|C7EYJ4|C7EYJ4_NARPS | 3.00E-48 | *Chalcone synthase* | *N. tazetta var. chinensis* |
| NODE_127772 | 140 | tr|Q5ZF91|Q5ZF91_PLAMJ | 3.00E-34 | *Chalcone synthase* | *P. alba (White poplar)* |
| NODE_656 | 119 | tr|Q9ZQS6|Q9ZQS6_IPOBA | 5.00E-17 | *Chalcone synthase* | *Lilium hybrid division I* |
| NODE_3044 | 146 | tr|G1FET0|G1FET0_PAELC | 1.00E-05 | *Chalcone synthase (Naregenin-chalcone synthase)* | *P. americana (Avocado)* |
| NODE_9993 | 105 | tr|I1IMN5|I1IMN5_BRADI | 2.00E-09 | *Chalcone synthase* | *B. rapa var. purpuraria* |
| NODE_4513 | 190 | tr|G7IN58|G7IN58_MEDTR | 1.00E-08 | *Chalcone synthase* | *M. aurantiacus (Orange bush monkey flower) (Diplacus aurantiacus)* |
| NODE_55588 | 320 | sp|Q7XA39|RGA4_SOLBU | 3.00E-19 | *Chalcone synthase 1* | *S. lycopersicum (Tomato) (Lycopersicon esculentum)* |
| NODE_18992 | 463 | tr|H6UJ36|H6UJ36_CAMSI | 2.00E-58 | *Chalcone synthase 1* | *C. arborescens (Siberian pea tree)* |
| NODE_183495 | 1396 | tr|A8Q8J7|A8Q8J7_BRUMA | 2.00E-08 | *Chalcone synthase 2* | *C. chekiangoleosa* |
| NODE_25910 | 201 | tr|A8PZN1|A8PZN1_BRUMA | 2.00E-12 | *Chalcone synthase 2* | *C. chekiangoleosa* |
| NODE_29638 | 1154 | tr|B9RUA7|B9RUA7_RICCO | 7.00E-20 | *Chalcone synthase 3 (Naringenin-chalcone synthase 3)* | *R. graveolens (Common rue)* |
| NODE_33546 | 433 | tr|B9SN62|B9SN62_RICCO | 7.00E-76 | *Chalcone synthase 3 (Naringenin-chalcone synthase 3)* | *R. graveolens* |
| NODE_122333 | 460 | tr|Q84TM3|Q84TM3_ALLCE | 2.00E-55 | *Chalcone synthase B* | *A. cepa* |
| NODE_134083 | 584 | tr|Q84TM3|Q84TM3_ALLCE | 4.00E-42 | *Chalcone synthase B* | *A. cepa* |
| NODE_156026 | 314 | tr|Q84TM3|Q84TM3_ALLCE | 1.00E-96 | *Chalcone synthase B* | *A. cepa* |
| NODE_97001 | 167 | tr|O82416|O82416_PAPSO | 2.00E-39 | *Chalcone synthase J (Naringenin-chalcone synthase J)* | *P. hybrida* |
| NODE_127619 | 146 | sp|Q6QHK0|CFI_ALLCE | 2.00E-18 | *Chalcone--flavonone isomerase (Chalcone isomerase)* | *A. cepa* |
| NODE_17902 | 744 | sp|Q6QHK0|CFI_ALLCE | 4.00E-14 | *Chalcone--flavonone isomerase (Chalcone isomerase)* | *A. cepa* |
| NODE_45303 | 159 | sp|Q6QHK0|CFI_ALLCE | 2.00E-27 | *Chalcone--flavonone isomerase (Chalcone isomerase)* | *A. cepa* |
| NODE_106681 | 359 | tr|B3TLS6|B3TLS6_ELAGV | 5.00E-14 | *Chalcone-flavanone isomerase* | *Elaeis guineensis var. tenera (Oil palm)* |
| NODE_156395 | 1092 | tr|B3TLS6|B3TLS6_ELAGV | 8.00E-55 | *Chalcone-flavanone isomerase* | *E. guineensis var. tenera* |
| NODE_6432 | 145 | tr|B3TLU6|B3TLU6_ELAGV | 7.00E-126 | *Chalcone-flavanone isomerase* | *E. oleifera (American oil palm) (Corozo oleifera)* |
| NODE_145909 | 272 | tr|Q9LM78|Q9LM78_ARATH | 2.00E-06 | *Chalcone-flavanone isomerase family protein* | *A. thaliana* |
| NODE_94774 | 835 | tr|B6ETT7|B6ETT7_ARATH | 2.00E-43 | *Chalcone-flavanone isomerase family protein* | *A. thaliana* |
| NODE_97214 | 117 | tr|I1MXN9|I1MXN9_SOYBN | 2.00E-25 | *Cinnamate-4-hydroxylase* | *G. arboreum (Tree cotton) (G. nanking)* |
| NODE_178065 | 315 | tr|M8AMW7|M8AMW7_TRIUA | 8.00E-10 | *Cinnamic acid 4-hydroxylase* | *Verbena hybrida (Garden vervain) (V. hortensis)* |
| NODE_52437 | 1565 | tr|C7E5X8|C7E5X8_CAPAN | 2.00E-22 | *Cinnamoyl-CoA reductase* | *Cenchrus purpureus* |
| NODE_126336 | 140 | tr|O78327|O78327_CAPAN | 0 | *Cinnamoyl-CoA reductase* | *C. purpureus* |
| NODE_128146 | 146 | tr|B9RSN1|B9RSN1_RICCO | 2.00E-20 | *Cinnamoyl-CoA reductase* | *R. communis* |
| NODE_18506 | 242 | tr|B9RSN1|B9RSN1_RICCO | 2.00E-23 | *Cinnamoyl-CoA reductase* | *R. communis* |
| NODE_48985 | 222 | tr|B9RMR9|B9RMR9_RICCO | 2.00E-15 | *Cinnamoyl-CoA reductase* | *R. communis* |
| NODE_112414 | 210 | tr|B9SN32|B9SN32_RICCO | 8.00E-32 | *Cinnamoyl-CoA reductase* | *R. communis* |
| NODE_141792 | 176 | tr|O65366|O65366_ANTMA | 2.00E-12 | *Cinnamoyl-CoA reductase family* | *A. lyrata subsp. lyrata (Lyre-leaved rock-cress)* |
| NODE_133594 | 556 | tr|Q677C9|Q677C9_HYAOR | 1.00E-20 | *Cyanidin 3-O-rutinoside 5-O-glucosyltransferase (Ih5GT (Anthocyanin 5-O-glucosyltransferase) (Cyanidin-3-rhamnosylglucoside 5-O-glucosyltransferase) (Uridine diphosphoglucose-cyanidin 3-rhamnosylglucoside 5-O-glucosyltransferase)* | *Iris hollandica (Dutch iris)* |
| NODE_101378 | 204 | tr|Q9SI75|Q9SI75_ARATH | 1.00E-95 | *Flavone 3'-O-methyltransferase 1 (AtOMT1) (Caffeate O-methyltransferase 1) (Quercetin 3'-O-methyltransferase 1)* | *A. thaliana* |
| NODE_163477 | 125 | tr|Q9LW53|Q9LW53_ARATH | 3.00E-06 | *Flavone 3'-O-methyltransferase 1 (AtOMT1) (Caffeate O-methyltransferase 1) (Quercetin 3'-O-methyltransferase 1)* | *A. thaliana* |
| NODE_79716 | 184 | tr|Q677C9|Q677C9_HYAOR | 1.00E-07 | *Flavone synthase II* | *I. hollandica* |
| NODE_47298 | 241 | tr|Q677H2|Q677H2_HYAOR | 5.00E-26 | *Flavone synthase II* | *I. hollandica* |
| NODE_25929 | 772 | tr|Q5YJP8|Q5YJP8_HYAOR | 1.00E-25 | *Flavone synthase II* | *I. hollandica* |
| NODE_88540 | 184 | tr|B9SBI2|B9SBI2_RICCO | 2.00E-48 | *Flavonoid 3-hydroxylase* | *R. communis* |
| NODE_140442 | 658 | tr|Q6Q625|Q6Q625_WHEAT | 7.00E-15 | *Flavonoid 3' hydroxylase* | *T. urartu (Red wild einkorn) (Crithodium urartu)* |
| NODE_143139 | 568 | tr|D3KVP3|D3KVP3_WHEAT | 6.00E-90 | *Flavonoid 3' hydroxylase* | *T. urartu (Red wild einkorn) (Crithodium urartu)* |
| NODE_1415 | 549 | tr|G8GKP4|G8GKP4_TRIMO | 6.00E-86 | *Flavonoid 3' hydroxylase* | *Vanda coerulea* |
| NODE_1417 | 132 | tr|G8GKP4|G8GKP4_TRIMO | 1.00E-61 | *Flavonoid 3' hydroxylase* | *V. coerulea* |
| NODE_78589 | 340 | tr|F1SW38|F1SW38_9CARY | 1.00E-06 | *Flavonoid 3' hydroxylase* | *Tricyrtis hirta (Toad lily) (T. japonica)* |
| NODE_24408 | 617 | tr|Q5MG99|Q5MG99_IPOBA | 1.00E-10 | *Flavonoid 3'-hydroxylase* | *Lilium hybrid division I* |
| NODE_26575 | 158 | tr|F6HBC4|F6HBC4_VITVI | 5.00E-53 | *Flavonoid 3'-hydroxylase* | *V. vinifera* |
| NODE_107922 | 280 | tr|F6H335|F6H335_VITVI | 8.00E-15 | *Flavonoid 3'-hydroxylase (Fragment)* | *V. vinifera* |
| NODE_111470 | 271 | tr|Q7XJ49|Q7XJ49_ALLCE | 3.00E-13 | *Flavonoid glucosyl-transferase* | *A. cepa* |
| NODE_124890 | 266 | tr|Q7XJ50|Q7XJ50_ALLCE | 1.00E-12 | *Flavonoid glucosyl-transferase* | *A. cepa* |
| NODE_135882 | 590 | tr|Q7XJ49|Q7XJ49_ALLCE | 8.00E-23 | *Flavonoid glucosyl-transferase* | *A. cepa* |
| NODE_135884 | 370 | tr|Q7XJ49|Q7XJ49_ALLCE | 3.00E-74 | *Flavonoid glucosyl-transferase* | *A. cepa* |
| NODE_149468 | 127 | tr|Q7XJ49|Q7XJ49_ALLCE | 4.00E-41 | *Flavonoid glucosyl-transferase* | *A. cepa* |
| NODE_164789 | 207 | tr|Q7XJ52|Q7XJ52_ALLCE | 2.00E-16 | *Flavonoid glucosyl-transferase* | *A. cepa* |
| NODE_168947 | 107 | tr|Q7XJ50|Q7XJ50_ALLCE | 9.00E-12 | *Flavonoid glucosyl-transferase* | *A. cepa* |
| NODE_169683 | 210 | tr|Q7XJ49|Q7XJ49_ALLCE | 4.00E-07 | *Flavonoid glucosyl-transferase* | *A. cepa* |
| NODE_169783 | 194 | tr|Q7XJ51|Q7XJ51_ALLCE | 2.00E-13 | *Flavonoid glucosyl-transferase* | *A. cepa* |
| NODE_41143 | 593 | tr|Q7XJ50|Q7XJ50_ALLCE | 9.00E-07 | *Flavonoid glucosyl-transferase* | *A. cepa* |
| NODE_50404 | 194 | tr|Q7XJ50|Q7XJ50_ALLCE | 4.00E-61 | *Flavonoid glucosyl-transferase* | *A. cepa* |
| NODE_51012 | 229 | tr|Q7XJ49|Q7XJ49_ALLCE | 2.00E-13 | *Flavonoid glucosyl-transferase* | *A. cepa* |
| NODE_63722 | 112 | tr|Q7XJ52|Q7XJ52_ALLCE | 3.00E-22 | *Flavonoid glucosyl-transferase* | *A. cepa* |
| NODE_98052 | 374 | tr|Q7XJ49|Q7XJ49_ALLCE | 1.00E-07 | *Flavonoid glucosyl-transferase* | *A. cepa* |
| NODE_98693 | 145 | tr|Q7XJ50|Q7XJ50_ALLCE | 3.00E-32 | *Flavonoid glucosyl-transferase* | *A. cepa* |
| NODE_88488 | 127 | tr|F1DGA6|F1DGA6_COFAR | 2.00E-09 | *Flavonoid glucosyltransferase* | *Crocus sativus (Saffron)* |
| NODE_92261 | 673 | tr|F1DGA6|F1DGA6_COFAR | 1.00E-06 | *Flavonoid glucosyltransferase* | *C. sativus* |
| NODE_124892 | 319 | tr|B1Q468|B1Q468_ANTMA | 8.00E-45 | *Flavonoid glucoyltransferase UGT73E2* | *Antirrhinum majus (Garden snapdragon)* |
| NODE_147291 | 207 | tr|D1MZ08|D1MZ08_EUCGL | 8.00E-32 | *Flavonol 3-sulfotransferase (F3-ST)* | *Flaveria chlorifolia (Clasping yellowtops)* |
| CL1630Cntig1 | 176 | tr|B9T351|B9T351_RICCO | 2.00E-25 | *Flavonol 4'-sulfotransferase* | *R. communis* |
| NODE_140914 | 275 | tr|B9SKF9|B9SKF9_RICCO | 7.00E-140 | *Flavonol 4'-sulfotransferase* | *R. communis* |
| NODE_5123 | 221 | tr|B9SKF9|B9SKF9_RICCO | 1.00E-09 | *Flavonol 4'-sulfotransferase* | *R. communis* |
| NODE_110446 | 196 | tr|G7I9L9|G7I9L9_MEDTR | 5.00E-16 | *Flavonol sulfotransferase-like protein* | *M. truncatula (Barrel medic) (M. tribuloides)* |
| NODE_146536 | 1730 | tr|G7I9L9|G7I9L9_MEDTR | 3.00E-17 | *Flavonol sulfotransferase-like protein* | *M. truncatula* |
| NODE_177175 | 164 | tr|G7I9L9|G7I9L9_MEDTR | 2.00E-07 | *Flavonol sulfotransferase-like protein* | *M. truncatula* |
| NODE_64527 | 364 | tr|G7I9L9|G7I9L9_MEDTR | 5.00E-07 | *Flavonol sulfotransferase-like protein* | *M. truncatula* |
| NODE_14432 | 256 | tr|Q6DTI1|Q6DTI1_ALLCE | 9.00E-156 | *Flavonol synthase* | *A. cepa* |
| NODE_5129 | 314 | tr|B9SKF9|B9SKF9_RICCO | 6.00E-37 | *Flavonol synthase/flavanone 3-hydroxylase* | *R. communis* |
| NODE_5130 | 168 | tr|B9SKF9|B9SKF9_RICCO | 1.00E-89 | *Flavonol synthase/flavanone 3-hydroxylase* | *R. communis* |
| NODE_59264 | 233 | tr|B9SKF9|B9SKF9_RICCO | 2.00E-24 | *Flavonol synthase/flavanone 3-hydroxylase* | *R. communis* |
| NODE_59266 | 811 | tr|B9SKF9|B9SKF9_RICCO | 2.00E-06 | *Flavonol synthase/flavanone 3-hydroxylase* | *R. communis* |
| NODE_30685 | 273 | tr|B9T2G4|B9T2G4_RICCO | 3.00E-113 | *Flavonol synthase/flavanone 3-hydroxylase* | *R. communis* |
| NODE_48016 | 773 | tr|B9T2G4|B9T2G4_RICCO | 1.00E-17 | *Flavonol synthase/flavanone 3-hydroxylase* | *R. communis* |
| NODE_65000 | 190 | tr|B9T2G4|B9T2G4_RICCO | 3.00E-39 | *Flavonol synthase/flavanone 3-hydroxylase* | *R. communis* |
| NODE_102840 | 323 | tr|B9R7N2|B9R7N2_RICCO | 8.00E-58 | *Flavonol synthase/flavanone 3-hydroxylase* | *R. communis* |
| NODE_110399 | 129 | tr|B9R7N2|B9R7N2_RICCO | 8.00E-10 | *Flavonol synthase/flavanone 3-hydroxylase* | *R. communis* |
| NODE_46859 | 304 | tr|B9R7N2|B9R7N2_RICCO | 5.00E-88 | *Flavonol synthase/flavanone 3-hydroxylase* | *R. communis* |
| NODE_70738 | 191 | tr|B6UCC8|B6UCC8_MAIZE | 7.00E-77 | *Flavonol synthase/flavanone 3-hydroxylase* | *Z. mays* |
| NODE_114649 | 126 | tr|M8BFZ7|M8BFZ7_AEGTA | 2.00E-06 | *Isoflavone 2'-hydroxylase* | *A. tauschii (Tausch's goatgrass) (A. squarrosa)* |
| NODE_64095 | 368 | tr|G7ILT6|G7ILT6_MEDTR | 4.00E-25 | *Isoflavone 2'-hydroxylase* | *M. truncatula* |
| NODE_29528 | 155 | tr|B9SVE1|B9SVE1_RICCO | 6.00E-32 | *Isoflavone reductase* | *R. communis* |
| NODE_67198 | 431 | tr|B6SS11|B6SS11_MAIZE | 3.00E-06 | *Isoflavone reductase* | *Z. mays* |
| NODE_21627 | 2109 | tr|B6TKT5|B6TKT5_MAIZE | 4.00E-121 | *Isoflavone reductase homolog IRL* | *Z. mays* |
| NODE_95442 | 153 | tr|Q9FNN3|Q9FNN3_ARATH | 1.00E-11 | *Isoflavone reductase homolog P3* | *A. thaliana* |
| NODE_92982 | 161 | tr|D7UA79|D7UA79_VITVI | 1.00E-40 | *Isoflavone reductase-like protein 4* | *V. vinifera* |
| NODE_72259 | 386 | tr|Q5JKF8|Q5JKF8_ORYSJ | 1.00E-15 | *Os01g0735900 protein (Putative UDP-glycose:flavonoid glycosyltransferase) (Uncharacterized protein)* | *O. sativa subsp. japonica* |
| NODE_10439 | 262 | tr|Q6F325|Q6F325_ORYSJ | 9.00E-55 | *Os05g0578500 protein (Putative cinnamoyl CoA reductase)* | *O. sativa subsp. japonica* |
| NODE_36321 | 164 | tr|Q6K796|Q6K796_ORYSJ | 3.00E-19 | *Probable 4-coumarate--CoA ligase 2* | *O. sativa subsp. japonica* |
| NODE_37904 | 364 | tr|Q6K796|Q6K796_ORYSJ | 8.00E-48 | *Probable 4-coumarate--CoA ligase 2* | *O. sativa subsp. japonica* |
| NODE_164039 | 129 | tr|F6LR33|F6LR33_9ASPA | 4.00E-14 | *Putative flavonoid 3'-hydroxylase (Fragment)* | *Diospyros kaki (Kaki persimmon) (D. chinensis)* |
| NODE_134988 | 112 | tr|Q8LPV4|Q8LPV4_DESAN | 1.00E-15 | *Putative flavonoid 3'-hydroxylase (Fragment)* | *D. kaki (Kaki persimmon) (D. chinensis)* |
| NODE_94898 | 177 | tr|Q8W314|Q8W314_ORYSJ | 6.00E-45 | *Putative flavonoid glucosyl-transferase* | *O. sativa subsp. japonica* |
| NODE_60456 | 124 | tr|G7K0K8|G7K0K8_MEDTR | 4.00E-13 | *UDP-glucose flavonoid 3-O-glucosyltransferase* | *M. truncatula (Barrel medic) (M. tribuloides)* |
| NODE_93652 | 611 | tr|Q4VG19|Q4VG19_LILLO | 4.00E-50 | *UDP-glucose flavonoid glucosyl-transferase (Fragment)* | *Litchi chinensis (Lychee)* |
| NODE_70478 | 1115 | tr|M7Y5C0|M7Y5C0_TRIUA | 3.00E-29 | *UDP-glycose:flavonoid glycosyltransferase* | *Vigna mungo (Black gram) (Phaseolus mungo)* |
| NODE_123876 | 139 | tr|O82074|O82074_TROMA | 2.00E-13 | *UDP-glycose:flavonoid glycosyltransferase* | *V. mungo* |
| CL1781Cntig1 | 292 | tr|F4HTY0|F4HTY0_ARATH | 2.00E-21 | *UDP-glycosyltransferase 73C6 (Flavonol-3-O-glycoside-7-O-glucosyltransferase 1) (Zeatin O-glucosyltransferase 2)* | *A. thaliana* |
| NODE_118532 | 282 | tr|F4HTY0|F4HTY0_ARATH | 8.00E-16 | *UDP-glycosyltransferase 78D2 (Anthocyanin 3-O-glucosyltransferase) (Flavonol 3-O-glucosyltransferase (UDP glucose:flavonoid 3-O-glucosyltransferase)* | *A. thaliana* |
| NODE_118538 | 140 | tr|F4HTY0|F4HTY0_ARATH | 2.00E-28 | *UDP-glycosyltransferase 78D2 (Anthocyanin 3-O-glucosyltransferase) (Flavonol 3-O-glucosyltransferase.) (UDP glucose:flavonoid 3-O-glucosyltransferase)* | *A. thaliana* |
| NODE_174218 | 119 | tr|F6HAE8|F6HAE8_VITVI | 4.00E-61 | *UDP-sugar flavonoid glycosyltransferase* | *V. vinifera* |
| NODE_172510 | 146 | tr|C0KHJ9|C0KHJ9_9CARY | 2.00E-07 | *UDPG-flavonoid 3-O-glucosyl transferase* | *Tripsacum dactyloides (Gama grass) (Coix dactyloides)* |
