# Supplementary material for: De novo Transcriptome Analysis Revealed Genes Involved in Flavonoid and Vitamin C Biosynthesis in Phyllanthus emblica (L.)
Source: Front Plant Sci. 2016 Oct 27;7:1610. doi: 10.3389/fpls.2016.01610 (PMC5081490; doi:10.3389/fpls.2016.01610)
Supplement: Supplementary Table S5 — Annotated cytochrome P450s (CYPs) in P. emblica transcriptome. [file Table5.DOC]

**Supplementary table S5: Annotated cytochrome P450s (CYPs) in *P. emblica* transcriptome**

| **CONTIG** | **CYP450** |
| --- | --- |
| NODE_156687_length_148_cov_3.000000 | ABA 8-oxidase |
| NODE_57891_length_406_cov_1.534483 | allene oxide synthase |
| NODE_57892_length_214_cov_3.841121 | allene oxide synthase |
| NODE_57893_length_244_cov_3.000000 | allene oxide synthase |
| NODE_141173_length_1101_cov_6.000000 | cinammate 4-hydroxylase |
| CL2274Contig1 | cytochrome P450 |
| CL2272Contig1 | cytochrome P450 |
| NODE_103493_length_174_cov_2.000000 | cytochrome P450 |
| NODE_106721_length_173_cov_3.000000 | cytochrome P450 |
| NODE_108623_length_69_cov_3.000000 | cytochrome P450 |
| NODE_109957_length_117_cov_2.854701 | cytochrome P450 |
| NODE_118922_length_213_cov_12.000000 | Cytochrome P450 |
| NODE_118929_length_213_cov_3.000000 | Cytochrome P450 |
| NODE_11980_length_1310_cov_12.763359 | cytochrome P450 |
| NODE_139339_length_245_cov_4.000000 | cytochrome P450 |
| NODE_145387_length_97_cov_7.000000 | cytochrome P450 |
| NODE_176106_length_89_cov_3.000000 | cytochrome p450 |
| NODE_24747_length_359_cov_8.000000 | cytochrome P450 |
| NODE_35474_length_468_cov_5.576923 | cytochrome P450 |
| NODE_35476_length_206_cov_2.422330 | cytochrome P450 |
| NODE_35477_length_142_cov_2.809859 | cytochrome P450 |
| NODE_36605_length_578_cov_2.996540 | cytochrome P450 |
| NODE_47583_length_210_cov_2.980952 | cytochrome P450 |
| NODE_47584_length_152_cov_2.973684 | cytochrome P450 |
| NODE_5685_length_671_cov_3.000000 | cytochrome P450 |
| NODE_58735_length_225_cov_1.982222 | cytochrome P450 |
| NODE_6231_length_226_cov_3.000000 | cytochrome P450 |
| NODE_6779_length_466_cov_9.000000 | cytochrome P450 |
| NODE_73492_length_192_cov_2.468750 | cytochrome P450 |
| NODE_73881_length_195_cov_1.025641 | cytochrome P450 |
| NODE_85639_length_888_cov_2.989865 | cytochrome P450 |
| NODE_90238_length_624_cov_2.881410 | Cytochrome P450 |
| NODE_95761_length_145_cov_3.000000 | cytochrome P450 71A18 |
| NODE_113311_length_93_cov_2.000000 | cytochrome P450 71B13 |
| NODE_81178_length_426_cov_8.000000 | Cytochrome P450 78A11 |
| NODE_145396_length_999_cov_6.000000 | cytochrome P450 98A2 |
| NODE_169940_length_1002_cov_5.000000 | cytochrome P450 CYP709C1 |
| NODE_132180_length_412_cov_3.000000 | cytochrome P450 CYP71D177 |
| NODE_24602_length_1698_cov_9.000000 | cytochrome P450 CYP97A16 |
| NODE_87889_length_455_cov_2.643956 | cytochrome P450 ent-kaurene oxidase |
| NODE_115371_length_76_cov_2.000000 | Cytochrome P450 family protein, expressed |
| NODE_118920_length_147_cov_12.000000 | Cytochrome P450 family protein, expressed |
| NODE_152927_length_132_cov_1.992424 | Cytochrome P450 family protein, expressed |
| NODE_20029_length_170_cov_2.776471 | Cytochrome P450 family protein, expressed |
| NODE_50354_length_296_cov_2.081081 | Cytochrome P450 monooxygenase CYP704G7 |
| NODE_92562_length_175_cov_3.000000 | Cytochrome P450 monooxygenase CYP704G7 |
| NODE_73976_length_194_cov_1.855670 | cytochrome P450 monooxygenase CYP71D64 |
| NODE_58043_length_117_cov_2.000000 | Cytochrome P450 monooxygenase CYP72A61 |
| NODE_11978_length_362_cov_13.000000 | cytochrome P450, family 704, subfamily A, polypeptide 2 |
| NODE_74347_length_118_cov_2.593220 | cytochrome P450, family 78, subfamily A, polypeptide 7 |
| NODE_10288_length_134_cov_7.000000 | cytochrome P450, family 96, subfamily A, polypeptide 15 |
| NODE_42956_length_369_cov_4.975610 | cytochrome P450, family 96, subfamily A, polypeptide 5 |
| NODE_107011_length_111_cov_3.000000 | cytochrome P450, putative |
| NODE_118923_length_73_cov_15.000000 | cytochrome P450, putative |
| NODE_139899_length_88_cov_3.000000 | cytochrome P450, putative |
| NODE_164423_length_447_cov_3.000000 | cytochrome P450, putative |
| NODE_24502_length_628_cov_2.990446 | cytochrome P450, putative |
| NODE_35791_length_112_cov_1.000000 | cytochrome P450, putative |
| NODE_6781_length_296_cov_7.361486 | cytochrome P450, putative |
| NODE_7244_length_214_cov_6.000000 | cytochrome P450, putative |
| NODE_95951_length_130_cov_3.000000 | cytochrome P450, putative |
| NODE_145386_length_166_cov_10.000000 | cytochrome P450, putative, expressed |
| NODE_58862_length_360_cov_3.000000 | ent-kaurene oxidase |
| NODE_181444_length_160_cov_3.000000 | ent-kaurene oxidase, putative |
| NODE_65765_length_222_cov_2.382883 | fatty acid hydroperoxide lyase |
| NODE_24503_length_365_cov_3.783562 | flavone synthase II |
| NODE_183301_length_113_cov_2.946903 | flavonoid 3'-hydroxylase |
| NODE_50786_length_522_cov_2.630268 | flavonoid 3'-hydroxylase |
| NODE_175733_length_95_cov_2.736842 | flavonoid-3'-hydroxylase |
| NODE_99581_length_764_cov_2.917539 | glutamine synthetase |
| NODE_145395_length_144_cov_3.000000 | p-coumarate 3-hydroxylase |
| NODE_89663_length_187_cov_2.545455 | PREDICTED: 4-hydroxyphenylacetaldehyde oxime monooxygenase-like |
| NODE_11982_length_227_cov_10.000000 | PREDICTED: cytochrome P450 704C1-like |
| NODE_11983_length_529_cov_7.000000 | PREDICTED: cytochrome P450 704C1-like |
| NODE_177180_length_175_cov_4.000000 | PREDICTED: cytochrome P450 704C1-like |
| NODE_31076_length_669_cov_7.197309 | PREDICTED: cytochrome P450 71A1-like |
| NODE_59067_length_460_cov_2.282609 | PREDICTED: cytochrome P450 71A1-like |
| NODE_73006_length_82_cov_2.000000 | PREDICTED: cytochrome P450 71A1-like |
| NODE_30239_length_415_cov_3.739759 | PREDICTED: cytochrome P450 71D11-like |
| NODE_140853_length_115_cov_1.826087 | PREDICTED: cytochrome P450 734A6-like |
| NODE_17416_length_1577_cov_3.932150 | PREDICTED: cytochrome P450 734A6-like |
| NODE_58042_length_370_cov_4.948648 | PREDICTED: cytochrome P450 734A6-like |
| NODE_56633_length_316_cov_1.838608 | PREDICTED: cytochrome P450 734A6-like isoform 1 |
| NODE_7278_length_1394_cov_6.000000 | PREDICTED: cytochrome P450 734A6-like isoform 1 |
| NODE_174164_length_385_cov_4.979221 | PREDICTED: cytochrome P450 77A3-like |
| NODE_81180_length_264_cov_8.000000 | PREDICTED: cytochrome P450 78A4 |
| NODE_47582_length_913_cov_2.993428 | PREDICTED: cytochrome P450 83B1-like |
| NODE_72835_length_102_cov_1.980392 | PREDICTED: cytochrome P450 84A1-like |
| NODE_74725_length_1345_cov_4.991078 | PREDICTED: cytochrome P450 85A |
| NODE_35878_length_291_cov_2.439862 | PREDICTED: cytochrome P450 85A-like |
| NODE_19317_length_686_cov_2.997085 | PREDICTED: cytochrome P450 86A2-like |
| NODE_38584_length_527_cov_2.003795 | PREDICTED: cytochrome P450 86B1 |
| NODE_10286_length_299_cov_7.000000 | PREDICTED: cytochrome P450 86B1-like |
| NODE_144986_length_339_cov_5.000000 | PREDICTED: cytochrome P450 86B1-like |
| NODE_145391_length_536_cov_3.774254 | PREDICTED: cytochrome P450 86B1-like |
| NODE_58268_length_439_cov_6.047836 | PREDICTED: cytochrome P450 86B1-like |
| NODE_122215_length_1856_cov_3.001078 | PREDICTED: cytochrome P450 87A3-like |
| NODE_132588_length_323_cov_8.000000 | PREDICTED: cytochrome P450 89A2-like |
| NODE_67392_length_165_cov_1.969697 | PREDICTED: cytochrome P450 89A2-like |
| NODE_36960_length_254_cov_2.322835 | PREDICTED: cytochrome P450 89A2-like isoform 1 |
| NODE_104749_length_430_cov_2.648837 | PREDICTED: cytochrome P450 90B1-like |
| NODE_133_length_1408_cov_11.996449 | PREDICTED: cytochrome P450 90B1-like |
| NODE_165822_length_75_cov_3.000000 | PREDICTED: cytochrome P450 90B1-like |
| NODE_64536_length_541_cov_2.609982 | PREDICTED: cytochrome P450 90B1-like |
| NODE_64574_length_242_cov_2.132231 | PREDICTED: cytochrome P450 93A1-like |
| NODE_166408_length_119_cov_3.000000 | PREDICTED: cytochrome P450 94A1-like |
| NODE_27622_length_614_cov_5.889251 | PREDICTED: cytochrome P450 94A1-like |
| NODE_27995_length_932_cov_2.906652 | PREDICTED: cytochrome P450 94A1-like |
| NODE_67466_length_121_cov_1.561983 | PREDICTED: cytochrome P450 94A1-like |
| NODE_131767_length_1118_cov_2.992844 | PREDICTED: cytochrome P450 97B2, chloroplastic |
| NODE_29372_length_471_cov_2.609342 | PREDICTED: ent-kaurenoic acid oxidase 1-like |
| NODE_41916_length_419_cov_2.971360 | PREDICTED: ent-kaurenoic acid oxidase 1-like |
| NODE_41917_length_741_cov_3.819163 | PREDICTED: ent-kaurenoic acid oxidase 1-like |
| NODE_57698_length_825_cov_3.003636 | PREDICTED: ent-kaurenoic acid oxidase 1-like |
| NODE_90870_length_126_cov_2.269841 | PREDICTED: ent-kaurenoic acid oxidase 1-like |
| NODE_109411_length_152_cov_2.000000 | PREDICTED: isoflavone 2'-hydroxylase-like |
| NODE_169931_length_97_cov_6.000000 | PREDICTED: isoflavone 2'-hydroxylase-like |
| NODE_65983_length_333_cov_2.420420 | PREDICTED: LOW QUALITY PROTEIN: cytochrome P450 71A1-like |
| NODE_107728_length_251_cov_2.912351 | PREDICTED: LOW QUALITY PROTEIN: cytochrome P450 76C4-like |
| NODE_145389_length_121_cov_4.000000 | PREDICTED: LOW QUALITY PROTEIN: cytochrome P450 86B1-like |
| NODE_161710_length_95_cov_1.000000 | PREDICTED: LOW QUALITY PROTEIN: secologanin synthase-like |
| NODE_35792_length_198_cov_2.409091 | PREDICTED: LOW QUALITY PROTEIN: secologanin synthase-like |
| NODE_87991_length_95_cov_10.000000 | PREDICTED: LOW QUALITY PROTEIN: secologanin synthase-like |
| NODE_20590_length_812_cov_5.844828 | PREDICTED: phenylalanine N-monooxygenase-like |
| NODE_154161_length_185_cov_10.000000 | PREDICTED: secologanin synthase-like |
| NODE_87992_length_820_cov_11.000000 | PREDICTED: secologanin synthase-like |
| NODE_171939_length_575_cov_4.000000 | PREDICTED: uncharacterized protein LOC100248387 |
| NODE_39281_length_1063_cov_3.000000 | PREDICTED: uncharacterized protein LOC100248387 |
| NODE_145291_length_311_cov_5.000000 | putative constitutive photomorphogenesis and dwarfism protein |
| NODE_114005_length_109_cov_3.559633 | putative cytochrome P450 |
| NODE_154162_length_539_cov_12.000000 | putative cytochrome P450 |
| NODE_27624_length_381_cov_4.750656 | putative cytochrome P450 |
| NODE_64004_length_222_cov_2.405406 | Putative cytochrome P450 |
| NODE_168884_length_192_cov_3.000000 | senescence-associated protein 3 |
| NODE_50321_length_616_cov_2.595779 | senescence-associated protein 3 |
| NODE_6454_length_1079_cov_9.000000 | steroid 22-alpha hydroxylase |
| NODE_72186_length_443_cov_2.616253 | taxane 13-alpha-hydroxylase precursor |
| CL177Contig1 | AC068924_22 putative cytochrome P450 |
| NODE_6775_length_907_cov_9.000000 | AC073556_35 putative plant cytochrome P-450 protein |
| NODE_98819_length_743_cov_4.973082 | AF140609_1 cytochrome P450 CYP79E1 |
| NODE_179765_length_72_cov_3.000000 | C71E1_SORBI RecName: Full=4-hydroxyphenylacetaldehyde oxime monooxygenase; AltName: Full=Cytochrome P450 71E1 |
| NODE_7246_length_543_cov_11.414365 | C7D55_HYOMU RecName: Full=Premnaspirodiene oxygenase; Short=HPO; AltName: Full=Cytochrome P450 71D55 |
| NODE_113348_length_70_cov_2.000000 | alcohol dehydrogenase; AltName: Full=Germacrene A hydroxylase |
| NODE_15886_length_195_cov_12.000000 | probable obtusifoliol 14-alpha-demethylase CYP51 - wheat (fragment) |
| NODE_27417_length_418_cov_5.899521 | TCMO_PHAAU RecName: Full=Trans-cinnamate 4-monooxygenase; AltName: Full=Cinnamic acid 4-hydroxylase; Short=C4H; Short=CA4H; AltName: Full=Cytochrome P450 73; AltName: Full=Cytochrome P450C4H |
