# Supplementary material for: De novo Transcriptome Analysis Revealed Genes Involved in Flavonoid and Vitamin C Biosynthesis in Phyllanthus emblica (L.)
Source: Front Plant Sci. 2016 Oct 27;7:1610. doi: 10.3389/fpls.2016.01610 (PMC5081490; doi:10.3389/fpls.2016.01610)
Supplement: Supplementary Table S6 — Annotated glycosyltransferases (GTs) in P. Emblica transcriptome. [file Table6.DOC]

**Supplementary table S6: Annotated glycosyltransferases (GTs) in *P. emblica* transcriptome.**

| **CONTIG ID** | **GENE SIMILARITY** |
| --- | --- |
| NODE_139695_length_603_cov_7.898839 | anthocyanidin 3-O-glucosyltransferase |
| NODE_28251_length_101_cov_1.633663 | Cytokinin-O-glucosyltransferase |
| NODE_156091_length_478_cov_3.217573 | cytokinin-O-glucosyltransferase 3 |
| NODE_149468_length_338_cov_6.000000 | flavonoid glucosyl-transferase |
| NODE_164789_length_95_cov_2.663158 | flavonoid glucosyl-transferase |
| NODE_22303_length_550_cov_8.000000 | flavonoid glucosyl-transferase |
| NODE_50404_length_561_cov_2.709447 | flavonoid glucosyl-transferase |
| NODE_98693_length_342_cov_2.654971 | flavonoid glucosyl-transferase |
| NODE_61453_length_210_cov_1.771429 | flavonoid glucosyltransferase |
| NODE_142278_length_227_cov_2.991189 | glucosyltransferase 2 |
| NODE_137577_length_440_cov_5.000000 | hypothetical protein OsI_21986 |
| NODE_69427_length_133_cov_3.000000 | hypothetical protein SORBIDRAFT_02g022260 |
| CL1884Contig1 | hypothetical protein SORBIDRAFT_05g023250 |
| NODE_126132_length_142_cov_9.000000 | hypothetical protein SORBIDRAFT_06g033023 |
| NODE_139694_length_156_cov_6.000000 | hypothetical protein SORBIDRAFT_10g006140 |
| NODE_60530_length_76_cov_3.000000 | hypothetical protein SORBIDRAFT_10g023120 |
| NODE_131047_length_99_cov_4.000000 | hypothetical protein VITISV_004870 |
| NODE_22204_length_203_cov_2.940887 | Os01g0597800 |
| NODE_60534_length_188_cov_3.000000 | Os01g0697100 |
| NODE_119282_length_1134_cov_3.014109 | Os01g0805500 |
| NODE_105155_length_87_cov_2.000000 | Os02g0755900 |
| NODE_61182_length_164_cov_2.384146 | Os02g0755900 |
| NODE_151572_length_482_cov_5.000000 | Os04g0451200 |
| NODE_124892_length_641_cov_6.000000 | predicted protein |
| NODE_131667_length_1260_cov_3.000000 | predicted protein |
| NODE_180874_length_154_cov_2.000000 | predicted protein |
| NODE_181933_length_72_cov_3.000000 | predicted protein |
| NODE_28252_length_153_cov_2.843137 | predicted protein |
| NODE_37322_length_72_cov_5.000000 | predicted protein |
| NODE_38978_length_460_cov_2.995652 | predicted protein |
| NODE_103324_length_428_cov_2.852804 | PREDICTED: abscisate beta-glucosyltransferase |
| NODE_37325_length_106_cov_4.688679 | PREDICTED: anthocyanidin 5,3-O-glucosyltransferase-like |
| NODE_69429_length_138_cov_2.688406 | PREDICTED: hydroquinone glucosyltransferase-like |
| NODE_66756_length_502_cov_2.679283 | PREDICTED: LOW QUALITY PROTEIN: UDP-glycosyltransferase 85A1-like |
| NODE_160370_length_386_cov_3.000000 | PREDICTED: putative UDP-rhamnose:rhamnosyltransferase 1 |
| NODE_26146_length_211_cov_4.928910 | PREDICTED: UDP-glycosyltransferase 73B3-like |
| NODE_61643_length_376_cov_2.526596 | PREDICTED: UDP-glycosyltransferase 73B4-like |
| NODE_98049_length_268_cov_3.000000 | PREDICTED: UDP-glycosyltransferase 73B4-like |
| NODE_89298_length_69_cov_4.000000 | PREDICTED: UDP-glycosyltransferase 73C5-like |
| NODE_60218_length_778_cov_2.997429 | PREDICTED: UDP-glycosyltransferase 85A2 |
| NODE_112187_length_91_cov_2.747253 | PREDICTED: UDP-glycosyltransferase 85A2 isoform 1 |
| NODE_115842_length_376_cov_9.609042 | PREDICTED: UDP-glycosyltransferase 85A2 isoform 1 |
| NODE_41030_length_111_cov_2.000000 | PREDICTED: UDP-glycosyltransferase 85A2-like |
| NODE_95733_length_482_cov_2.904564 | PREDICTED: UDP-glycosyltransferase 85A2-like |
| NODE_115186_length_70_cov_2.000000 | PREDICTED: UDP-glycosyltransferase 85A3-like |
| NODE_47607_length_376_cov_2.824468 | PREDICTED: UDP-glycosyltransferase 85A3-like |
| NODE_76040_length_104_cov_1.817308 | PREDICTED: UDP-glycosyltransferase 87A2-like |
| NODE_28669_length_175_cov_2.428571 | PREDICTED: UDP-glycosyltransferase 92A1-like |
| NODE_70248_length_141_cov_2.553191 | PREDICTED: UDP-glycosyltransferase 92A1-like |
| NODE_46765_length_299_cov_2.755853 | putative glucosyl transferase |
| NODE_44559_length_208_cov_3.961539 | tetrahydroxychalcone glucosyltransferase |
| NODE_47528_length_711_cov_2.774965 | UDP glucosyltransferase |
| NODE_154920_length_1099_cov_4.000000 | UDP glycosyltransferase |
| NODE_134264_length_195_cov_4.558974 | UDP-glucose glucosyltransferase |
| NODE_169701_length_209_cov_3.000000 | UDP-glucose glucosyltransferase |
| NODE_46806_length_668_cov_2.928144 | UDP-glucose glucosyltransferase |
| NODE_38112_length_419_cov_1.539379 | UDP-glucose:glucosyltransferase |
| NODE_51011_length_176_cov_3.653409 | UDP-glucose:isoflavone 7-O-glucosyltransferase |
| NODE_109506_length_184_cov_2.972826 | UDP-glucosyltransferase 73B2 |
| NODE_92750_length_473_cov_2.985201 | UDP-glucosyltransferase HvUGT5876 |
| NODE_70182_length_345_cov_2.356522 | UDP-glucosyltransferase, putative |
| NODE_72756_length_342_cov_2.602339 | UDP-glucosyltransferase, putative |
| NODE_91648_length_162_cov_3.592592 | UDP-glucosyltransferase, putative |
| NODE_25604_length_242_cov_3.363636 | UDP-glucuronosyltransferase |
