# Supplementary material for: De novo Transcriptome Analysis Revealed Genes Involved in Flavonoid and Vitamin C Biosynthesis in Phyllanthus emblica (L.)
Source: Front Plant Sci. 2016 Oct 27;7:1610. doi: 10.3389/fpls.2016.01610 (PMC5081490; doi:10.3389/fpls.2016.01610)
Supplement: Supplementary Table S7 — Details of all the genes involved in vitamin C biosynthesis identified in P. emblica transcriptome. [file Table7.DOCX]

| **Supplementary Table S7: Details of all the transcripts identified of genes involved in vitamin C biosynthesis** | | | |
| --- | --- | --- | --- |
| **Gene Name** | **Contig ID** | **Sequence Length (bp)** | **BLASTX Analysis** |
| *Hexokinase* | NODE_110516_length_78_cov_3.000000 | 110 | gi\|209978718\|gb\|ACJ04704.1\| hexokinase 1 |
|  | NODE_128573_length_205_cov_11.000000 | 237 | gi\|356520703\|ref\|XP_003529000.1\| PREDICTED: hexokinase-1-like |
|  | NODE_128575_length_165_cov_11.000000 | 197 | gi\|356495748\|ref\|XP_003516735.1\| PREDICTED: hexokinase-2-like |
|  | NODE_128577_length_143_cov_14.000000 | 175 | gi\|224143653\|ref\|XP_002325031.1\| predicted protein |
|  | NODE_128578_length_406_cov_11.000000 | 438 | gi\|11066213\|gb\|AAG28503.1\|AF196966_1 hexokinase |
|  | NODE_128582_length_406_cov_3.000000 | 438 | gi\|11066213\|gb\|AAG28503.1\|AF196966_1 hexokinase |
|  | NODE_128584_length_596_cov_3.000000 | 628 | gi\|356513171\|ref\|XP_003525287.1\| PREDICTED: hexokinase-1-like |
|  | NODE_128588_length_141_cov_3.000000 | 173 | gi\|224143653\|ref\|XP_002325031.1\| predicted protein |
|  | NODE_137345_length_72_cov_1.000000 | 104 | gi\|112430755\|gb\|ABI18156.1\| hexokinase |
|  | NODE_163387_length_294_cov_3.000000 | 326 | gi\|224083000\|ref\|XP_002306924.1\| predicted protein |
|  | NODE_165359_length_264_cov_3.000000 | 296 | gi\|75291596\|sp\|Q6Q8A5.1\|HXK2_TOBAC RecName: Full=Hexokinase-2, chloroplastic; AltName: Full=NtHxK2; Flags: Precursor |
|  | NODE_21524_length_89_cov_12.000000 | 121 | gi\|11066213\|gb\|AAG28503.1\|AF196966_1 hexokinase |
|  | NODE_21537_length_660_cov_4.000000 | 692 | gi\|225436573\|ref\|XP_002274759.1\| PREDICTED: hexokinase-3 |
|  | NODE_21539_length_463_cov_4.000000 | 495 | gi\|147820969\|emb\|CAN74594.1\| hypothetical protein VITISV_003476 |
|  | NODE_25586_length_1670_cov_3.000000 | 1702 | gi\|225445080\|ref\|XP_002283608.1\| PREDICTED: hexokinase-1-like |
|  | NODE_56438_length_84_cov_1.595238 | 116 | gi\|357132912\|ref\|XP_003568072.1\| PREDICTED: LOW QUALITY PROTEIN: hexokinase-5-like |
|  | NODE_58981_length_239_cov_3.933054 | 271 | gi\|356513171\|ref\|XP_003525287.1\| PREDICTED: hexokinase-1-like |
|  | NODE_58982_length_193_cov_2.512953 | 225 | gi\|224059148\|ref\|XP_002299739.1\| predicted protein |
|  | NODE_65758_length_100_cov_3.000000 | 132 | gi\|18026821\|gb\|AAL55635.1\|AF118134_1 hexokinase-related protein 1 |
|  | NODE_65759_length_83_cov_2.975904 | 115 | gi\|357165897\|ref\|XP_003580531.1\| PREDICTED: hexokinase-4, chloroplastic-like |
|  | NODE_65760_length_86_cov_4.767442 | 118 | gi\|255538922\|ref\|XP_002510526.1\| hexokinase, putative |
|  | NODE_67061_length_139_cov_3.000000 | 171 | gi\|209978720\|gb\|ACJ04705.1\| hexokinase 2 |
|  | NODE_76044_length_215_cov_2.944186 | 247 | gi\|225457987\|ref\|XP_002275922.1\| PREDICTED: hexokinase-2, chloroplastic |
| *Phosphoglucose isomerase* | NODE_30602_length_69_cov_2.000000 | 101 | gi\|2588818\|dbj\|BAA23182.1\| cytosolic phosphoglucose isomerase |
|  | NODE_119675_length_683_cov_4.000000 | 715 | gi\|22775491\|dbj\|BAC11914.1\| cytosolic phosphoglucose isomerase |
|  | NODE_147422_length_1651_cov_5.000000 | 1683 | gi\|356508007\|ref\|XP_003522754.1\| PREDICTED: glucose-6-phosphate isomerase-like |
|  | NODE_21889_length_168_cov_5.000000 | 200 | gi\|194702654\|gb\|ACF85411.1\| unknown |
|  | NODE_119673_length_1027_cov_4.000000 | 1059 | gi\|225458305\|ref\|XP_002282774.1\| PREDICTED: glucose-6-phosphate isomerase, cytosolic 1 |
| *Phosphomannose isomerase* | NODE_67580_length_144_cov_2.277778 | 176 | gi\|60617303\|gb\|AAX31279.1\| phosphomannose isomerase |
|  | NODE_74537_length_110_cov_2.527273 | 142 | gi\|30679394\|ref\|NP_192229.3\| uncharacterized protein |
|  | NODE_146077_length_1143_cov_2.995625 | 1175 | gi\|147841344\|emb\|CAN60179.1\| hypothetical protein VITISV_011365 |
|  | NODE_185515_length_68_cov_3.000000 | 100 | gi\|255572106\|ref\|XP_002526993.1\| mannose-6-phosphate isomerase, putative |
|  | NODE_50845_length_1243_cov_2.938053 | 1275 | gi\|115434298\|ref\|NP_001041907.1\| Os01g0127900 |
| *Phosphomanno-mutase* | NODE_130010_length_1566_cov_6.000000 | 1598 | gi\|255584011\|ref\|XP_002532751.1\| phosphoglucomutase, putative |
|  | NODE_22412_length_821_cov_4.907430 | 853 | gi\|242074812\|ref\|XP_002447342.1\| hypothetical protein SORBIDRAFT_06g033280 |
| *GDP-D-Mannose Pyrophosphorylase* | NODE_25547_length_102_cov_8.000000 | 134 | gi\|222876022\|gb\|ACM69043.1\| GDP-D-mannose pyrophosphorylase |
|  | NODE_25578_length_126_cov_3.000000 | 158 | gi\|113952525\|gb\|ABI48955.1\| GDP-D-mannose pyrophosphorylase |
|  | NODE_35211_length_471_cov_8.000000 | 503 | gi\|225457154\|ref\|XP_002283703.1\| PREDICTED: mannose-1-phosphate guanyltransferase alpha isoform 1 |
|  | NODE_35213_length_167_cov_3.000000 | 199 | gi\|359488770\|ref\|XP_003633817.1\| PREDICTED: mannose-1-phosphate guanyltransferase alpha-like isoform 2 |
|  | NODE_70329_length_256_cov_2.699219 | 288 | gi\|255577159\|ref\|XP_002529463.1\| conserved hypothetical protein |
|  | NODE_9996_length_845_cov_11.000000 | 877 | gi\|326532794\|dbj\|BAJ89242.1\| predicted protein |
|  | NODE_138975_length_402_cov_5.000000 | 434 | gi\|225457154\|ref\|XP_002283703.1\| PREDICTED: mannose-1-phosphate guanyltransferase alpha isoform 1 |
|  | NODE_138980_length_190_cov_4.000000 | 222 | gi\|26006499\|gb\|AAN77308.1\| Putative GDP-mannose pyrophosphorylase |
|  | NODE_145729_length_99_cov_3.000000 | 131 | gi\|80973464\|gb\|ABB53473.1\| GDP-mannose pyrophosphorylase |
|  | NODE_25550_length_96_cov_5.000000 | 128 | gi\|80973464\|gb\|ABB53473.1\| GDP-mannose pyrophosphorylase |
|  | NODE_35215_length_346_cov_2.898844 | 378 | gi\|148841127\|gb\|ABR14736.1\| GDP-mannose pyrophosphorylase |
|  | NODE_25577_length_153_cov_3.000000 | 185 | gi\|357460959\|ref\|XP_003600761.1\| Mannose-1-phosphate guanyltransferase |
|  | NODE_25582_length_233_cov_2.888412 | 265 | gi\|224831509\|gb\|ACN66754.1\| GMP |
|  | NODE_35205_length_189_cov_9.000000 | 221 | gi\|224135729\|ref\|XP_002322146.1\| predicted protein |
|  | NODE_35209_length_493_cov_8.517241 | 525 | gi\|224135729\|ref\|XP_002322146.1\| predicted protein |
|  | NODE_25545_length_234_cov_8.166667 | 266 | gi\|224112691\|ref\|XP_002316262.1\| predicted protein |
|  | NODE_25555_length_236_cov_8.000000 | 268 | gi\|148909316\|gb\|ABR17757.1\| unknown |
|  | NODE_25559_length_173_cov_20.000000 | 205 | gi\|357460959\|ref\|XP_003600761.1\| Mannose-1-phosphate guanyltransferase |
|  | NODE_25564_length_147_cov_23.000000 | 179 | gi\|357125854\|ref\|XP_003564604.1\| PREDICTED: probable mannose-1-phosphate guanylyltransferase 3-like |
|  | NODE_25565_length_361_cov_20.000000 | 393 | gi\|225449380\|ref\|XP_002282422.1\| PREDICTED: mannose-1-phosphate guanylyltransferase 1 isoform 1 |
|  | NODE_25572_length_256_cov_3.000000 | 288 | gi\|226493137\|ref\|NP_001142302.1\| uncharacterized protein LOC100274471 |
|  | NODE_25573_length_108_cov_3.000000 | 140 | gi\|15233308\|ref\|NP_191118.1\| mannose-1-phosphate guanylyltransferase |
| *GDP-Mannose-3ʹ, 5ʹ-epimerase* | NODE_134516_length_300_cov_17.000000 | 332 | gi\|146432257\|gb\|ABQ41112.1\| GDP-mannose-3',5'-epimerase |
|  | NODE_25299_length_297_cov_4.000000 | 329 | gi\|146432257\|gb\|ABQ41112.1\| GDP-mannose-3',5'-epimerase |
|  | NODE_134515_length_311_cov_17.000000 | 343 | gi\|255580957\|ref\|XP_002531297.1\| dtdp-glucose 4-6-dehydratase, putative |
|  | NODE_134517_length_90_cov_19.000000 | 122 | gi\|356573081\|ref\|XP_003554693.1\| PREDICTED: GDP-mannose 3,5-epimerase 1-like |
|  | NODE_134519_length_99_cov_17.000000 | 131 | gi\|82400136\|gb\|ABB72807.1\| NAD-dependent epimerase/dehydratase family protein-like protein |
|  | NODE_134521_length_123_cov_17.000000 | 155 | gi\|218117843\|dbj\|BAH03299.1\| GDP-D-mannose-3',5'-epimerase |
|  | NODE_134523_length_91_cov_17.000000 | 123 | gi\|212275446\|ref\|NP_001130997.1\| uncharacterized protein LOC100192102 |
|  | NODE_184707_length_88_cov_3.000000 | 120 | gi\|284437921\|gb\|ADB85573.1\| GDP-D-mannose 3',5'-epimerase |
|  | NODE_25295_length_176_cov_3.795455 | 208 | gi\|115482032\|ref\|NP_001064609.1\| Os10g0417600 |
|  | NODE_25297_length_276_cov_4.000000 | 308 | gi\|195620882\|gb\|ACG32271.1\| GDP-mannose 3,5-epimerase 1 |
|  | NODE_25301_length_539_cov_3.686456 | 571 | gi\|223469963\|gb\|ACM90324.1\| GDP-D-mannose-3',5'-epimerase |
|  | NODE_147465_length_105_cov_2.000000 | 137 | gi\|356573081\|ref\|XP_003554693.1\| PREDICTED: GDP-mannose 3,5-epimerase 1-like |
| *GDP-L-galactose phosphorylase / L-Galactose Guanyltransferase* | NODE_781_length_1188_cov_6.989899 | 1220 | gi\|357157220\|ref\|XP_003577725.1\| PREDICTED: GDP-L-galactose phosphorylase 1-like |
|  | NODE_9993_length_114_cov_8.000000 | 146 | gi\|357157220\|ref\|XP_003577725.1\| PREDICTED: GDP-L-galactose phosphorylase 1-like |
|  | NODE_123747_length_232_cov_21.000000 | 264 | gi\|357156527\|ref\|XP_003577487.1\| PREDICTED: GDP-L-galactose phosphorylase 1-like |
| *L-Galactose-1-P Phosphatase* | NODE_131521_length_357_cov_2.994398 | 389 | gi\|319739583\|gb\|ADV59926.1\| putative L-galactose-1-phosphate phosphatase |
|  | NODE_15054_length_159_cov_9.000000 | 191 | gi\|55275406\|gb\|AAV49506.1\| L-galactose-1-phosphate phosphatase |
|  | NODE_15056_length_147_cov_9.000000 | 179 | gi\|55275406\|gb\|AAV49506.1\| L-galactose-1-phosphate phosphatase |
|  | NODE_15060_length_561_cov_5.937612 | 593 | gi\|319739583\|gb\|ADV59926.1\| putative L-galactose-1-phosphate phosphatase |
|  | NODE_15063_length_147_cov_3.000000 | 179 | gi\|55275406\|gb\|AAV49506.1\| L-galactose-1-phosphate phosphatase |
|  | NODE_15065_length_159_cov_3.000000 | 191 | gi\|55275406\|gb\|AAV49506.1\| L-galactose-1-phosphate phosphatase |
|  | NODE_15068_length_112_cov_6.000000 | 144 | gi\|255581603\|ref\|XP_002531606.1\| myo inositol monophosphatase, putative |
| *L-Galactose Dehydrogenase* | NODE_14146_length_546_cov_8.007326 | 578 | gi\|146432259\|gb\|ABQ41113.1\| L-galactose dehydrogenase |
|  | NODE_14147_length_96_cov_6.000000 | 128 | gi\|307136456\|gb\|ADN34261.1\| L-galactose dehydrogenase |
| *L-Galactono-1, 4-lactone Dehydrogenase* | NODE_164775_length_195_cov_2.958974 | 227 | gi\|3986289\|dbj\|BAA34995.1\| L-Galactono-1,4-lactone dehydrogenase |
|  | NODE_58479_length_1611_cov_2.981999 | 1643 | gi\|218186410\|gb\|EEC68837.1\| hypothetical protein OsI_37417 |
